# Gut-specific telomerase expression counteracts systemic aging in telomerase-deficient zebrafish

**DOI:** 10.1038/s43587-023-00401-5

**Published:** 2023-05-04

**Authors:** Mounir El Maï, Malia Bird, Asma Allouche, Seniye Targen, Naz Şerifoğlu, Bruno Lopes-Bastos, Jean-Marie Guigonis, Da Kang, Thierry Pourcher, Jia-Xing Yue, Miguel Godinho Ferreira

**Affiliations:** 1 Institute for Research on Cancer and Aging of Nice (IRCAN), CNRS UMR7284, INSERM U1081, Université Côte d’Azur, Nice, France; 2 Instituto Gulbenkian de Ciência, Oeiras, Portugal; 3 Laboratory Transporter in Imaging and Radiotherapy in Oncology, Institut des Sciences du Vivant Frederic Joliot, Commissariat à l’Energie Atomique et aux Energies Alternatives, Université Côte d’Azur, Nice, France; 4 State Key Laboratory of Oncology in South China, Collaborative Innovation Center for Cancer Medicine, Sun Yat-sen University Cancer Center, Guangzhou, China

**Keywords:** Telomeres, Ageing, Senescence

## Abstract

Telomere shortening is a hallmark of aging and is counteracted by telomerase. As in humans, the zebrafish gut is one of the organs with the fastest rate of telomere decline, triggering early tissue dysfunction during normal zebrafish aging and in prematurely aged telomerase mutants. However, whether telomere-dependent aging of an individual organ, the gut, causes systemic aging is unknown. Here we show that tissue-specific telomerase expression in the gut can prevent telomere shortening and rescues premature aging of *tert*^−/−^. Induction of telomerase rescues gut senescence and low cell proliferation, while restoring tissue integrity, inflammation and age-dependent microbiota dysbiosis. Averting gut aging causes systemic beneficial impacts, rescuing aging of distant organs such as reproductive and hematopoietic systems. Conclusively, we show that gut-specific telomerase expression extends the lifespan of *tert*^−/−^ by 40%, while ameliorating natural aging. Our work demonstrates that gut-specific rescue of telomerase expression leading to telomere elongation is sufficient to systemically counteract aging in zebrafish.

## Abstract (web summary)

Telomere shortening is a hallmark of aging and genetic telomerase deficiency causes premature aging phenotypes and reduces lifespan. Here the authors show that gut-specific telomerase expression is sufficient to rescue aging phenotypes and extend the lifespan of telomerase-deficient zebrafish, as well as ameliorate signs of aging in wild-type animals.

## Main

The discovery that the lifespan can be genetically extended in *Caenorhabditis elegans* initiated a new era of research aiming to define interventions to promote the lifespan and healthspan extension^1^. Since then, improvements achieved by modulating the hallmarks of aging have provided specific therapeutic targets for healthy aging^2^. For example, reverting age-related deregulation of nutrient-sensing mechanisms by interventions such as caloric restriction or rapamycin (mammalian target of rapamycin (mTOR) inhibitor) treatment increases the lifespan in several species^3,4^. Similarly, genetic and pharmacological removal of senescent cells can delay age-associated defects resulting in lifespan extension in mice^5,6^.

Telomere shortening and dysfunction are major determinants of aging^2^. Telomeres protect chromosome ends from degradation and recognition by DNA damage response pathways^7^. Due to the ‘end-replication problem’, telomeres gradually shorten with each round of cell division. When telomeres are critically short, DNA damage responses are triggered that culminate in cell cycle arrest, replicative senescence^8,9^ and loss of tissue integrity^2^. Telomere shortening is counteracted by a specific reverse transcriptase termed telomerase. *TERT* expression, the catalytic component of telomerase, is restricted to stem or progenitor cells^10,11^. However, telomerase expression is insufficient to fully restore telomere erosion throughout the lifespan of vertebrates; consequently, aging organisms show signs of telomere dysfunction^11^.

Patients that carry mutations in telomerase or telomere maintenance protein genes show premature shortening of telomeres, short life expectancy and a set of pathologies known as telomere biology disorders (TBDs)^12,13^. Similarly, depletion of telomerase in zebrafish accelerates telomere shortening, causing premature aging phenotypes and reduced lifespan in *tert*^−/−^ animals^14–16^. *tert*^−/−^ zebrafish present the same dysfunction events observed during natural zebrafish aging at an anticipated rate^14–16^. DNA damage associated with short telomeres is first observed in the gut; it is concomitant with reduced cell proliferation, accumulation of senescent cells and functional defects both in naturally aging and *tert*^−/−^ zebrafish^14,17^. Importantly, telomere shortening results in cellular and functional defects in the gut at a time when other organs are clear of tissue dysfunction^14^. As in zebrafish, the human gastrointestinal system is one of the organs with the fastest rate of telomere shortening^18^. Severe TBDs are often associated with gastrointestinal syndromes^19,20^; increased telomere shortening was observed in the intestinal epithelium of patients with inflammatory bowel disease^21,22^. Therefore, gut homeostasis is heavily dependent on telomere integrity.

Over a century ago, Metchnikov proposed that loss of tissue integrity and aging derives from chronic systemic inflammation promoted by increased intestine permeability and infiltration of microorganisms and their products into the bloodstream. Even though weakening of the intestinal barrier is a major feature of gut aging^4^, it is unclear whether organ-specific decline influences overall organismal aging. In this Article, we show that gut-specific telomerase expression in *tert*^−/−^ zebrafish is sufficient to delay gut aging. Counteracting gut aging improves health of the entire organism, reverting gut microbiota dysbiosis and aging phenotypes in distant organs of *tert*^−/−^ zebrafish. Finally, we show that the most relevant systemic effect of gut-specific telomerase expression is lifespan extension, while improving natural aging. Thus, gut telomere-dependent aging controls aging of the entire organism.

## Results

### Tissue-specific telomerase expression rescues gut aging

**Fig. 1:**
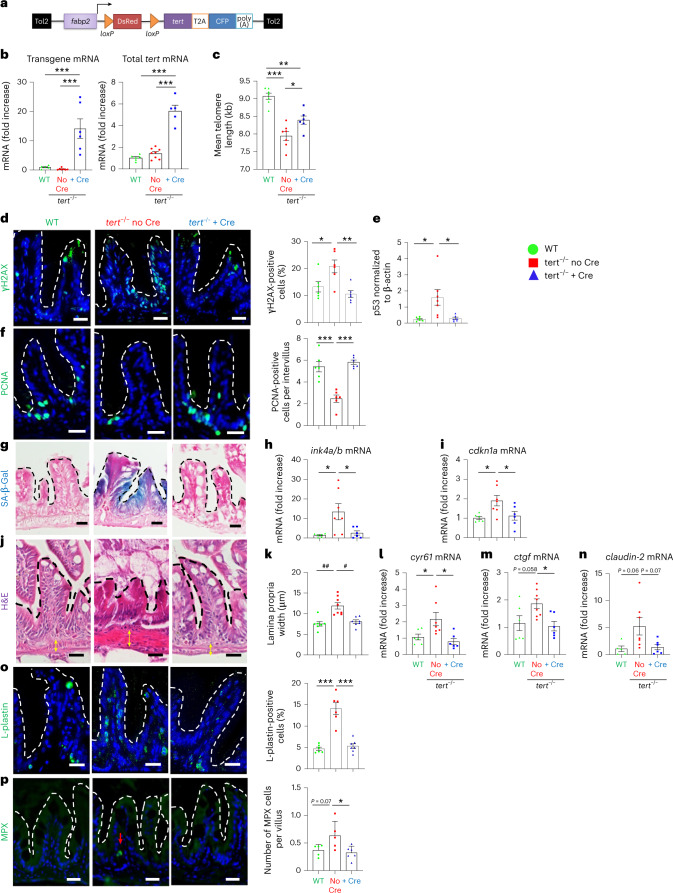
Gut-specific and Cre-mediated *tert* expression rescues gut aging phenotypes. **a**, Schematic representation of the transgene for Cre-inducible and tissue-specific expression of *tert* mRNA. **b**, RT–qPCR analysis of *tert* transgene mRNA and total *tert* mRNA (endogenous + transgene) expression in 9-month-old gut extracts (*n*_WT_ = 5 and 6; $n_{tert^{-/-}\, {\mathrm{no}}\, {\mathrm{Cre}}}$ = 7 and 8 and $n_{tert^{-/-}\, +\, {\mathrm{Cre}}}$ = 6 and 5 fish, respectively; levels were normalized by *rps11* gene expression levels). **c**, Quantification of mean telomere length by TRF analysis (*n*_WT_ = 7; $n_{tert^{-/-}\, {\mathrm{no}}\, {\mathrm{Cre}}}$ = 7 and $n_{tert^{-/-}\, +\, {\mathrm{Cre}}}$ = 6 fish). **d**, Representative immunofluorescence images of DNA damage staining (γH2AX; left) and quantification (right; *n*_WT_ = 6; $N_{tert^{-/-}\, {\mathrm{no}}\, {\mathrm{Cre}}}$ = 6 and $n_{tert^{-/-}\, +\, {\mathrm{Cre}}}$ = 6 fish). **e**, Quantification of p53 protein levels (normalized by β-actin) analyzed by western blot (*n*_WT_ = 6; $N_{tert^{-/-}\, {\mathrm{no}}\, {\mathrm{Cre}}}$ = 7 and $n_{tert^{-/-}\, +\, {\mathrm{Cre}}}$ = 6 fish). **f**, Representative immunofluorescence images of proliferation staining (left, proliferation cell nuclear antigen (PCNA)) and quantification (right, *n*_WT_ = 6; $n_{tert^{-/-}\, {\mathrm{no}}\, {\mathrm{Cre}}}$ = 6 and $n_{tert^{-/-}\, +\, {\mathrm{Cre}}}$ = 6 fish). **g**, Representative image of SA-β-Gal staining. **h**,**i**, RT–qPCR analysis of the senescence-associated genes *ink4a/b* (p15/16) (**h**) and *cdkn1a* (p21) (**i**) expression (*n*_WT_ = 6; $n_{tert^{-/-}\, {\mathrm{no}}\, {\mathrm{Cre}}}$ = 7 and $n_{tert^{-/-}\, +\, {\mathrm{Cre}}}$ = 6 fish). **j**,**k**, Representative hematoxylin and eosin (H&E)-stained sections of the gut (**j**). The yellow arrows delineate the lamina propria width quantified in **k** (*n*_WT_ = 7; $n_{tert^{-/-}\, {\mathrm{no}}\, {\mathrm{Cre}}}$ = 8 and $n_{tert^{-/-}\, +\, {\mathrm{Cre}}}$ = 7 fish). **l**,**m**, RT–qPCR analysis of the YAP target genes *cyr61* (**l)** and *ctgf* expression (**m**) (*n*_WT_ = 6; $n_{tert^{-/-}\, {\mathrm{no}}\, {\mathrm{Cre}}}$ = 8 and $n_{tert^{-/-}\, +\, {\mathrm{Cre}}}$ = 6 fish). **n**, RT–qPCR analysis of the junction protein-associated gene *claudin-2* expression (*n*_WT_ = 5; $n_{tert^{-/-}\, {\mathrm{no}}\, {\mathrm{Cre}}}$ = 7 and $n_{tert^{-/-}\, +\, {\mathrm{Cre}}}$ = 6 fish). **o**, Representative immunofluorescence images of immune cell staining (left, L-plastin) and quantification (right, *n*_WT_ = 6 fish; $n_{tert^{-/-}\, {\mathrm{no}}\, {\mathrm{Cre}}}$ = 6 fish and $n_{tert^{-/-}\, +\, {\mathrm{Cre}}}$ = 7 fish). **p**, Representative immunofluorescence images of neutrophil staining (left, myeloperoxidase (MPX)) and quantification (right, *n*_WT_ = 5 fish; $n_{tert^{-/-}\, {\mathrm{no}}\, {\mathrm{Cre}}}$ = 5 fish and $n_{tert^{-/-}\, +\, {\mathrm{Cre}}}$ = 6 fish). All analyses are based on 9-month-old fish gut sections or extracts. Scale bar, 20 µm. The dashed lines delineate the gut villi. All data are presented as the mean ± s.e.m. *P < 0.05, **P < 0.01, ***P < 0.001, using a one-way ANOVA and post hoc Tukey test; *P < 0.05, **P < 0.01, using a Kruskal–Wallis and post hoc Dunn test. Source data

To investigate how telomere-dependent gut aging impacts the organism, we generated a zebrafish transgenic line harboring a Cre-inducible zebrafish *tert* transgene driven by an enterocyte-specific *fabp2* promoter^23^ in a *tert*^+/−^ genetic background (Fig. 1a). After crossing this line with *tert*^+/−^ fish, we induced the *tert* transgene expression by microinjection of Cre mRNA in one-cell-stage embryos, creating the following sibling fish: (1) *tert*^−/−^ containing the full construct (*tert*^−/−^ no Cre); (2) *tert*^−/−^-expressing *tert* transgene (*tert*^−/−^ + Cre); and (3) *tert*^+/+^ containing the full construct (wild-type (WT)).

**Extended Data Fig. 1:**
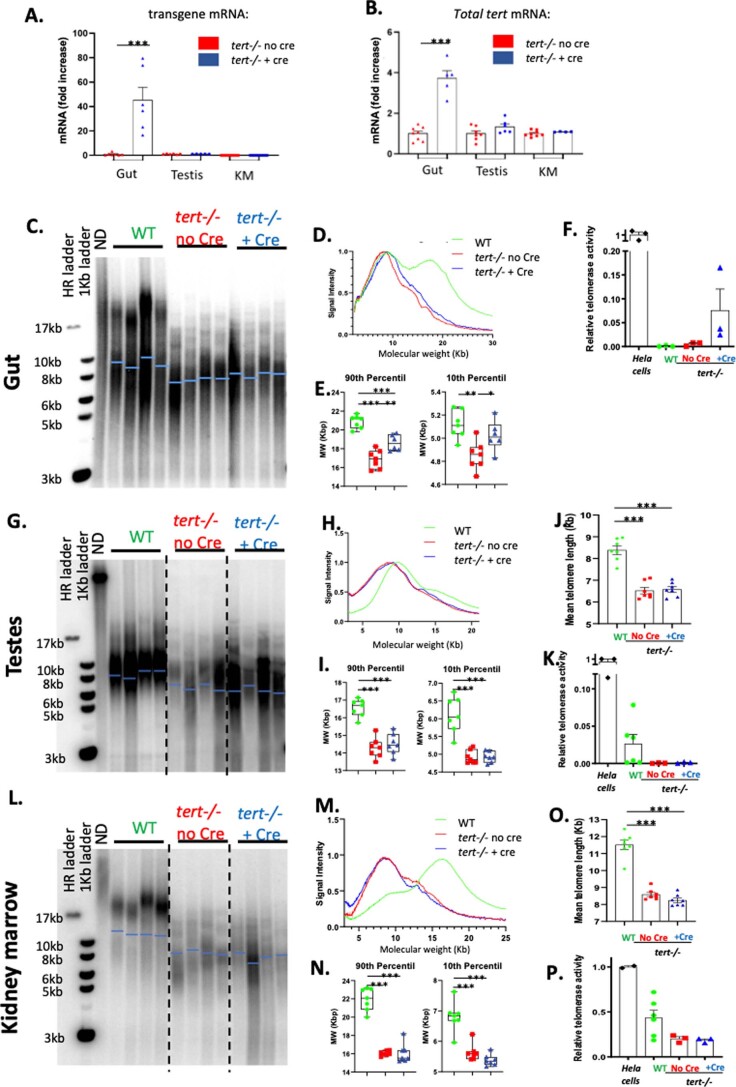
The *tert* transgene is specifically expressed in the gut resulting in telomerase activity and telomere elongation in the gut of tert^−/−^ zebrafish. **a, b**. RT-qPCR analysis of *tert* transgene mRNA (**A**.) and total *tert* mRNA (**B**.) expression in gut (N_tert−/− No Cre_=7 and 8 and N_tert−/− +Cre_=6 and 5 fish respectively), testes (N_tert−/− No Cre_=6 and 7 and N_tert−/− +Cre_=5 and 6 fish respectively) and kidney marrow (N_tert−/− No Cre_=9 and 8 and N_tert−/− +Cre_=7 and 4 fish respectively) extracts derived from 9-month-old fish. **c–e**. Telomere length analyses of genomic DNA extracted from 9-month-old gut samples (N_WT_=7, N_tert−/− No Cre_=7 and N_tert−/− +Cre_=6 fish). Representative images of TRF analysis (blue bars represents mean telomere length) **(C.)**, mean TRF densitometry curves (**D.)**, and quantification of median TRF of the longest (90^th^ percentile; left panel) and the shortest (10^th^ percentile; right panel) telomeres (**E.)**. **f**. Quantification of telomerase activity in gut of 12-month-old zebrafish using quantitative Telomerase Repeated Amplification Protocol (qTRAP) assay (N=3 fish per condition). Hela cell extracts were used as positive control for telomerase activity. **g–j**. Telomere length analyses of genomic DNA extracted from 9-month-old testes samples (N = 7 fish per condition). Representative images of TRF analysis (blue bars represents mean telomere length). Dashed lines delineate cropped parts of the same Southern blot image **(G.)**, mean TRF densitometry curves (**H.)**, quantification of median TRF of the longest (90^th^ percentile; left panel) and the shortest (10^th^ percentile; right panel) telomeres (**I.)** and quantification of mean telomere length (**J.)**. **k**. Quantification of telomerase activity in testes of 12-month-old zebrafish by qTRAP (N = 3 fish per condition except N_WT_ = 6). **l–o**. Telomere length analyses of genomic DNA extracted from 9-month-old kidney marrow samples (N = 7 fish per condition). Representative images of TRF analysis (blue bars represents mean telomere length)**(L.)**, mean TRF densitometry curves (**M.)**, quantification of median TRF of the longest (90^th^ percentile; left panel) and the shortest (10^th^ percentile; right panel) telomeres **(N.)** and quantification of mean telomere length **(O.). p**. Quantification of telomerase activity in kidney marrow of 12-month-old zebrafish using qTRAP assay (N = 3 fish per condition except N_WT_ = 6). Data are represented as mean +/−SEM (***p-value<0.001, using one-way ANOVA and post-hoc Tukey tests). Boxes of Tukey boxplots represent the median and interquartile range. Source data

As expected, we did not detect expression of the *tert* transgene in mock-injected fish, while Cre microinjection resulted in the excision of the STOP cassette and *tert* transgene expression (Fig. 1b, left). This led to an approximate fivefold enrichment of total *tert* mRNA (endogenous and transgene *tert* mRNA) in the gut of *tert*^−/−^ + Cre fish when compared to mock-injected control tissues (*tert*^−/−^ no Cre and WT; Fig. 1b, right). Consequently, we observed a higher telomerase activity in *tert*^−/−^ + Cre compared to both WT and *tert*^−/−^ no Cre (Extended Data Fig. 1f). To test whether expression of the *tert* transgene is sufficient to prevent telomere shortening, we performed telomere restriction fragment (TRF) analysis on gut samples of 9-month-old fish. As described previously^14,15^, we noted that the range of telomere length in the gut of WT fish exhibited a bimodal pattern (Extended Data Fig. 1c,d). This pattern reflects the differences in telomere length between cell types. The telomere length of WT blood cells was longer (approximately 19 kb) than other tissues (approximately 9 kb) leading to a two-peak densitometry pattern^14,15,24^. Reflecting the requirement of telomerase to sustain long telomeres in blood cells, the telomere length of *tert*^−/−^ blood cells was drastically reduced compared to WT (as seen by the loss of the longer peak; Extended Data Fig. 1d)^14,15^. Even though expression of *tert* complementary DNA (cDNA) driven by the *fabp2* promoter did not restore telomere length to WT levels, induction of the *tert* transgene was sufficient to elongate telomeres in the whole-gut tissues of *tert*^−/^^−^ + Cre fish (7.9–8.4 kb, *n* = 6–7, *P* < 0.05; Fig. 1c and Extended Data Fig. 1c,e). Like *tert*^−/−^ no Cre fish, *tert*^−/−^ + Cre fish lacked the longer telomere peak, indicating that the *tert* transgene is not expressed in blood cells. As described previously^14,15^, telomere shortening in the gut of *tert*^−/−^ no Cre fish leads to an increase in DNA damage, as observed by γH2AX immunofluorescence and p53 protein levels, when compared to WT fish (Fig. 1d,e). Consistent with telomere elongation, these markers are reverted by telomerase expression in the gut of *tert*^−/−^ + Cre fish. Thus, *tert* transgene expression is sufficient to counteract telomere dysfunction in the gut of *tert*^−/−^ fish by extending telomere length.

**Extended Data Fig. 2:**
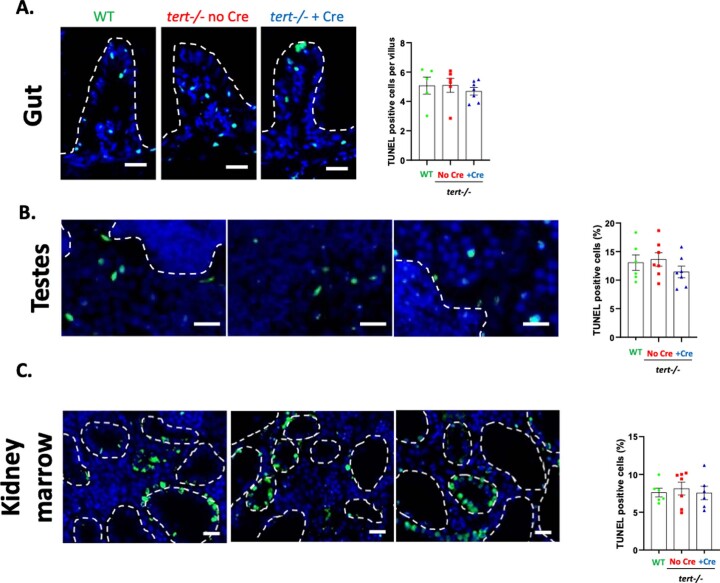
9-month-old fish do not exhibit differences in apoptosis between groups. At 9-months of age, no differences in apoptosis were detected in gut, testes and kidney marrow comparing *tert*^−/−^ No Cre, *tert*^−/−^ +Cre and WT fish. **a–c**. Representative immunofluorescence images of apoptotic cell staining (TUNEL assay; left panel) and quantification (right panel) in gut (**A.;** N_WT_ = 5, N_tert−/− No Cre_ = 6 and N_tert−/− +Cre_ = 7 fish), testes (**B.;** N_WT_ = 6, N_tert−/− No Cre_ = 7 and N_tert−/− +Cre_ = 7 fish) and kidney marrow (**C.;** N_WT_ = 6, N_tert−/− No Cre_ = 7 and N_tert−/− +Cre_ = 6 fish) tissues of 9-month-old zebrafish. Scale bar: 20µm. Dashed lines delineate gut villi (**A**.), mature spermatid area (**B**.), or kidney tubules (**C**.). All data are represented as mean +/- SEM (no significance was detected comparing all conditions and using one-way ANOVA and post-hoc Tukey tests). Source data

To test whether *tert* transgene expression can rescue the aging defects of telomerase-deficient animals, we analyzed the gut of 9-month-old fish. As observed previously^14,15^, the gut of *tert*^−/−^ no Cre fish showed reduced cell proliferation compared to WT fish. Enterocyte-specific telomerase expression rescued the proliferative capacity of this organ to WT levels (Fig. 1f). Senescence-associated β-galactosidase (SA-β-gal) assays and transcription levels of the senescence-associated genes *ink4a/b* (p15/16) and *cdkn1a* (p21) revealed that telomerase expression reduced cell senescence to WT levels (Fig. 1g–i). Consistent with our previous work^17^, we detected no differences in apoptosis in the gut of WT, *tert*^−/−^ no Cre and *tert*^−/−^ + Cre of 9-month-old fish (Extended Data Fig. 2a).

These cellular defects observed in *tert*^−/−^ fish impact tissue integrity^14,15,17^. We observed that *tert*^−/−^ no Cre fish exhibited morphological tissue defects with thickening of the lamina propria (Fig. 1j,k). Loss of intestinal barrier integrity led to activation of the Yes-associated protein (YAP) transcription factor responsible for tissue regeneration^25,26^. Consistent with loss of gut integrity, expression of the YAP target genes *cyr61* and *ctgf* was increased in *tert*^−/−^ no Cre fish (Fig. 1l,m). Likewise, *claudin-2* mRNA levels were higher in *tert*^−/−^ no Cre fish (Fig. 1n). Increased gene expression of the tight junction protein *claudin-2* occurs during primate aging and enhances in vivo intestinal permeability^27,28^. Strikingly, all these phenotypes were rescued in *tert*^−/^^−^ + Cre fish (Fig. 1j–n).

**Extended Data Fig. 3:**
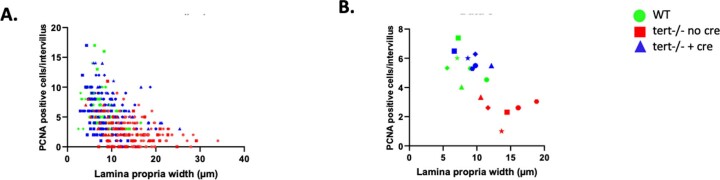
Proliferation in individual intervilli negatively correlates with local inflammation. Intervillus-based correlation plot between cell proliferation and gut *lamina propria* width in 9-month-old fish. Plots illustrate the correlation between the number of PCNA positive cells within each intervillus and lamina propria width below each respective intervillus (analyzed based on the immunofluorescence staining experiment of Fig. 1f). **a**. Each point represents a single intervillus analyzed from either WT, *tert*^−/−^ No Cre or *tert*^−/−^ +Cre. **b**. Each point represents a single zebrafish analyzed from either WT, *tert*^−/−^ No Cre or *tert*^−/−^ +Cre (N = 6 fish per condition). Source data

We observed that the number of proliferative cells in individual intervilli was negatively correlated with the thickness of the lamina propria (Extended Data Fig. 3). Plotting either individual intervilli (Extended Data Fig. 3a) or individual fish (Extended Data Fig. 3b), we noticed that WT and *tert*^−/−^ + Cre clustered separately from *tert*^−/−^ no Cre samples. In addition, we observed higher infiltration of total immune cells and neutrophils in the intestinal epithelium of the *tert*^−/−^ no Cre fish compared to WT (Fig. 1o,p). In line with a rescue of intestinal integrity, the number of immune cells was reverted to WT levels in *tert*^−/−^ + Cre fish. Considering that thickening of the gut lamina propria results from immune cell infiltrates, these results suggest that cell proliferation is locally affected by inflammation. Thus, rescuing tissue integrity promotes the proliferative capacity of the gut in part by reducing tissue inflammation.

### Local effects

#### Gut *tert* rescues gene expression and metabolism

**Fig. 2:**
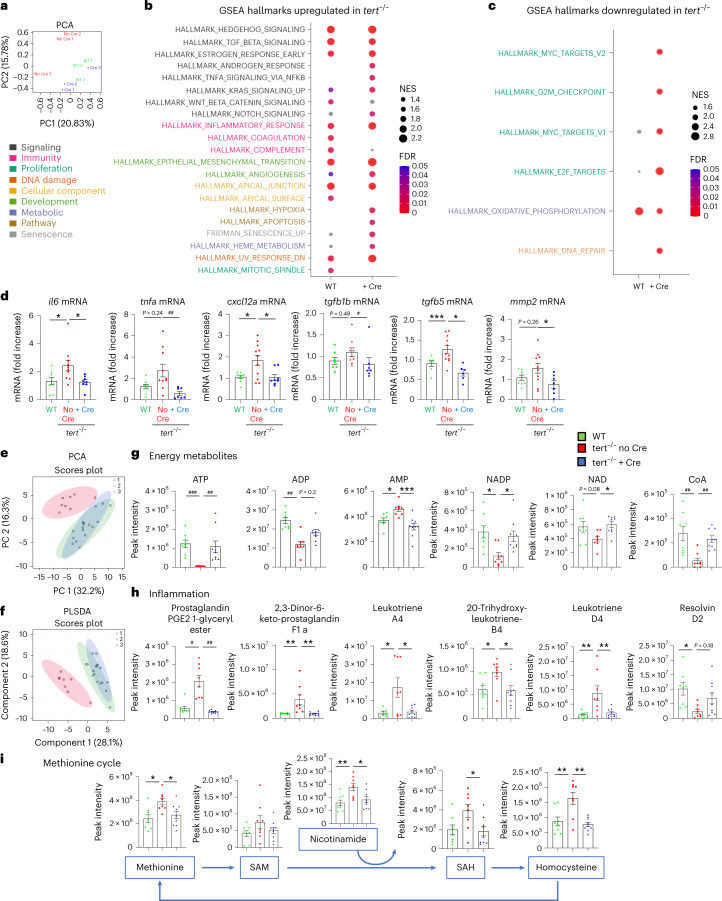
Gut-specific *tert* expression rescues gut transcriptomics and metabolomics profiles. **a**, Principal component analysis (PCA)-based on untargeted transcriptomics data of 9-month-old gut samples. A clustering between *tert*^−/−^ + Cre and WT was observed while the *tert*^−/−^ no Cre group was clearly distinguishable from *tert*^−/−^ no Cre fish (*n* = 3 per group). **b**,**c**, Identification of upregulated (**b**) or downregulated (**c**) hallmarks in *tert*^−/−^ no Cre compared to either WT or *tert*^−/−^ + Cre, based on GSEA. The normalized enrichment scores (NES) depict to what degree the pathway genes are overrepresented in WT or *tert*^−/−^ + Cre, compared to *tert*^−/−^ no Cre. Gene sets related to senescence, inflammation and morphogenesis were enriched while the hallmarks of proliferation and oxidative phosphorylation were downregulated in the gut of *tert*^−/−^ no Cre fish compared to the other two groups. **d**, RT–qPCR analysis of inflammation-related gene expression (*il6*, *tnfa*, *tgfb1b* and *tgfb5*) and SASP-related gene expression (*il6*, *tnfa*, cxcl12a, *tgfb1b*, *tgfb5* and *mmp2*) in 9-month-old gut samples (*n*_WT_ = 8 fish, $n_{tert^{-/-}\, {\mathrm{no}}\, {\mathrm{Cre}}}$ = 10 fish and $n_{tert^{-/-}\, +\, {\mathrm{Cre}}}$ = 8 fish for *il6;* *n*_WT_ = 8 fish, $n_{tert^{-/-}\, {\mathrm{no}}\, {\mathrm{Cre}}}$ = 9 fish and $n_{tert^{-/-}\, +\, {\mathrm{Cre}}}$ = 8 fish for *tnfa;* *n*_WT_ = 8 fish, $n_{tert^{-/-}\, {\mathrm{no}}\, {\mathrm{Cre}}}$ = 11 fish and $n_{tert^{-/-}\, +\, {\mathrm{Cre}}}$ = 8 fish for *cxcl12;* *n*_WT_ = 7 fish, $n_{tert^{-/-}\, {\mathrm{no}}\, {\mathrm{Cre}}}$ = 9 fish and $n_{tert^{-/-}\, +\, {\mathrm{Cre}}}$ = 7 fish for *tgfb1b;* *n*_WT_ = 7 fish, $n_{tert^{-/-}\, {\mathrm{no}}\, {\mathrm{Cre}}}$ = 11 fish and $n_{tert^{-/-}\, +\, {\mathrm{Cre}}}$ = 6 fish for *tgfb5;* and *n*_WT_ = 8 fish, $n_{tert^{-/-}\, {\mathrm{no}}\, {\mathrm{Cre}}}$ = 10 fish and $n_{tert^{-/-}\, +\, {\mathrm{Cre}}}$ = 7 fish for *mmp2*). **e**,**f**, PCA (**e**) and partial least squares discriminant analysis (PLS-DA) (**f**) clustering analysis based on untargeted metabolomics data of 9-month-old gut samples. A clustering between *tert*^−/−^ + Cre and WT was observed while the *tert*^−/−^ no Cre group was clearly distinguishable from the other (*n*_WT_ = 8 fish, $n_{tert^{-/-}\, {\mathrm{no}}\, {\mathrm{Cre}}}$ = 8 fish and $n_{tert^{-/-}\, +\, {\mathrm{Cre}}}$ = 9 fish). The score plot is presented with a confidence ellipse of 95%. **g**–**i**, Metabolomics analysis of energy metabolites (**g**), inflammatory metabolites (**h**) and methionine cycle pathway (**i**) in 9-month-old gut samples (*n*_WT_ = 8 fish, $n_{tert^{-/-}\, {\mathrm{no}}\, {\mathrm{Cre}}}$ = 8 fish and $n_{tert^{-/-}\, +\, {\mathrm{Cre}}}$ = 9 fish). All data are presented as the mean ± s.e.m.; *P < 0.05, **P < 0.01, ***P < 0.001, using a one-way ANOVA and post hoc Tukey test; *P < 0.05; *P < 0.01, ***P < 0.001, using a Kruskal–Wallis and post hoc Dunn test). Source data

By comparing the expression profiles of whole-gut tissues using RNA sequencing (RNA-seq), we observed a distinguishable transcriptomics signature in *tert*^−/−^ no Cre, while WT and *tert*^−/−^ + Cre clustered together (Fig. 2a and Supplementary Data). Gene set enrichment analysis (GSEA) showed that most of the hallmarks were similarly deregulated in *tert*^−/−^ no Cre than either WT or *tert*^−/−^ + Cre. The transcriptomics profiles of the *tert*^−/−^ no Cre gut are enriched in gene expression related to senescence, inflammation and morphogenesis (Fig. 2b), while the hallmarks of proliferation or oxidative phosphorylation are downregulated (Fig. 2c). We further validated this transcriptomics recovery of senescence-associated secretory phenotype (SASP)/inflammation-related genes by analyzing the transcription levels of the *il6*, *tnfa*, *cxcl12a*, *tgfb1b*, *tgfb5* and *mmp2* genes (Fig. 2d). In line with the previous results, these transcription profiles confirmed that telomerase expression rescued cell proliferation, loss of tissue integrity, senescence and inflammation seen in the gut of *tert*^−/−^ no Cre fish.

Changes in metabolism have been associated with aging and might reflect cellular defects, such as gradual mitochondrial dysfunction with age^29,30^. Consistently, we previously showed that 9-month-old *tert*^−/−^ gut exhibits mitochondrial dysfunction accompanied by low ATP and high reactive oxygen species (ROS) levels^17^. Unsupervised and supervised clustering analyses of metabolomics profiles revealed that both WT and *tert*^−/−^ + Cre samples clustered tightly while *tert*^−/−^ no Cre samples differed from the other groups (Fig. 2e,f and Extended Data Fig. 5a). Most metabolites were reduced (621) or enriched (141) in both WT and *tert*^−/−^ + Cre when compared to *tert*^−/−^ no Cre fish (Extended Data Fig. 5b). Consistent with our previous work^17^, we observed a drastic reduction of energetic metabolites, such as ATP, ADP, nicotinamide adenine dinucleotide (NAD), NAD phosphate (NADP) and coenzyme A (CoA), in *tert*^−/−^ no Cre fish (Fig. 2g). Following the anaerobic glycolysis pathway, we noticed lower levels of glucose-6-phosphate and fructose 1,6-bisphosphate and higher amounts of pyruvate and lactate (Extended Data Fig. 5c). Considering that glucose did not vary between groups, our results suggest that the gut of *tert*^−/−^ no Cre fish acquired higher levels of anaerobic glycolysis. We also detected higher pentose shunt activity in *tert*^−/−^ no Cre gut, evidenced by increased amounts of ribose-5-phosphate and erythrose 4-phosphate (Extended Data Fig. 5d). Except for citrate levels, all the detected metabolites of the citric acid cycle were elevated in *tert*^−/−^ no Cre fish (Extended Data Fig. 6a). Altogether, the gut energetic metabolism of *tert*^−/−^ no Cre fish were engaged in uncoupled oxidative phosphorylation, consistent with damaged mitochondria, low ATP levels and higher production of ROS. By expressing *tert* transgene in the gut, *tert*^−/−^ no Cre metabolic alterations were prevented in the entire tissue.

In line with our previous results depicting higher inflammation of *tert*^−/−^ no Cre fish, we observed an overall increase in arachidonic metabolism with higher levels of pro-inflammatory molecules, such as prostaglandins and leukotrienes (Fig. 2h). Consistently, we detected lower amounts of anti-inflammatory resolvin D2 in *tert*^−/−^ no Cre fish when compared to the other groups. Among the detected amino acids, methionine was significantly enriched in *tert*^−/−^ no Cre gut compared to the other genotypes (Fig. 2i). We also observed an overall increase in methionine metabolites in the mutant gut that might be allowed by higher levels of nicotinamides. The steroid pathway was also enriched in *tert*^−/−^ no Cre fish. Not only the stress hormone cortisol but also female hormones (such as 16-oxoestrone or estradiol) were elevated in *tert*^−/−^ no Cre male fish (Extended Data Fig. 6b). Overall, our unbiased metabolomics analysis described an altered metabolism profile in *tert*^−/−^ no Cre that was recovered by gut-specific telomerase expression.

### Local effects

#### Gut *tert* rescues gut microbiota dysbiosis

**Fig. 3:**
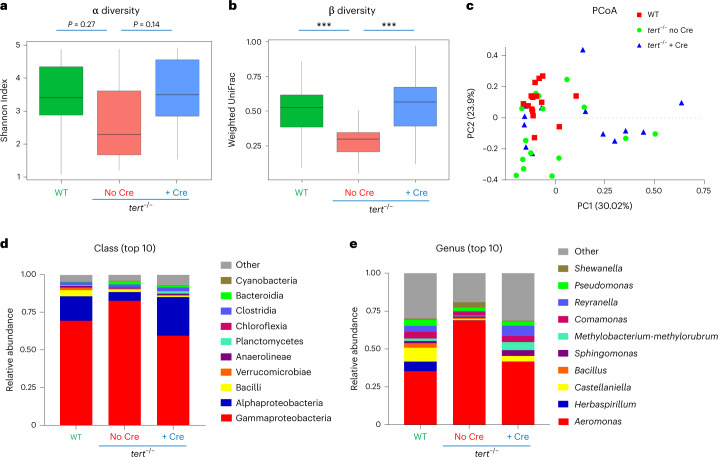
Gut-specific *tert* expression rescues gut microbiota dysbiosis. Telomere elongation in the gut of *tert*^−/−^ + Cre fish rescued gut microbiota composition and diversity to WT levels compared to *tert*^−/−^ no Cre fish, which exhibited gut microbiota dysbiosis. **a**, Quantification of microbiome α diversity (within samples) using the Shannon index (*P* values were determined using a two-sided Wilcoxon signed-rank test) in the gut of 9-month-old fish. **b**, Quantification of microbiome β diversity using weighted UniFrac distance (within groups; ***P < 0.001 using a two-sided Tukey test) in the gut of 9-month-old fish. **c**, PCoA of the β diversity distance (weighted UniFrac) in the gut of 9-month-old fish. **d**, Relative abundance of top 10 bacterial classes in the microbiome of the three different groups in the gut of 9-month-old fish. **e**, Relative abundance of top 10 bacterial *genera* in the microbiome of the three different groups in the gut of 9-month-old fish. For all the figures, *n*_WT_ = 15 fish, $n_{tert^{-/-}\, {\mathrm{no}}\, {\mathrm{Cre}}}$ = 15 fish and $n_{tert^{-/-}\, +\, {\mathrm{Cre}}}$ = 14 fish; α and β diversity data are shown as Tukey boxplots, where the boxes represent the median and interquartile range and the bars represent the minimum and maximum values. Source data

Gut microbiota dysbiosis is associated with a dysfunctional intestinal barrier and is suggested to generate a feed-forward loop involving gut permeability, inflammation and dysbiosis in aging^31,32^. However, it was unclear whether delaying gut aging would counteract gut microbiota dysbiosis. To investigate if telomerase expression in the gut of *tert*^−/−^ fish ameliorates gut dysbiosis, we performed high-throughput sequencing of the V3 and V4 regions of 16S ribosomal DNA of 9-month-old zebrafish gut. As described for human aging^33,34^, we observed diminished microbial diversity in *tert*^−/−^ no Cre when compared to WT controls. Both α (within samples) and β (within groups) analyses showed lower diversity in *tert*^−/−^ no Cre individuals compared to other groups (Fig. 3a,b). According to a reduced β diversity, using principal coordinates analysis (PCoA), we observed a clustering of *tert*^−/−^ no Cre samples while WT and *tert*^−/−^ + Cre samples were more dispersed (Fig. 3c).

The relative abundance of bacterial taxonomic units at the class level revealed an overall alteration of gut microbiota composition in *tert*^−/−^ no Cre fish that was recovered by *tert* expression (Fig. 3d). At the class level, we observed in the *tert*^−/−^ no Cre group a decreased abundance of Alphaproteobacteria and Planctomycetes along with an enrichment in Gammaproteobacteria, Bacteroidia and Fibrobacteria (Fig. 3d and Extended Data Fig. 7a). While Alphaproteobacteria inhibit host cell death and promote proliferation^35^, Gammaproteobacteria expansion is associated with early age-dependent loss of intestinal barrier integrity in flies^32^. Similarly, at the genus level, the Alphaproteobacteria *Reyranella* and *Defluviimonas* were reduced while the Gammaproteobacteria *Aeromonas* and *Shewanella* along with *Bacteroides*, a Bacteroidia-related genus, were enriched in *tert*^−/−^ no Cre fish (Fig. 3e and Extended Data Fig. 7b). Both *Shewanella* and *Aeromonas* have been described as deleterious in humans, with *Shewanella* causing intra-abdominal infections^36^ and *Aeromonas* being associated with inflammatory bowel disease and inflammation^37,38^. Within the *Aeromonas* genus, *Aeromonas veronii* was strikingly overrepresented in *tert*^−/−^ no Cre fish (Extended Data Fig. 7c). From the Bacteroidia class, *Bacteroides uniformis*, *Parabacteroides merdae* and *Bacteroides ovatus* were similarly enriched in *tert*^−/−^ no Cre and are considered ‘pathobionts’ that profit from a dysregulated environment to overtake commensal symbionts and become pathogenic^39–41^. Overall, our analysis of gut microbiota composition revealed a wrongly balanced gut microbiome in *tert*^−/−^ no Cre fish, containing a less diverse bacterial community with increased representation of otherwise pathogenic taxa microbiota being more pathogenic. These features were reverted by gut-specific telomerase expression.

### Systemic effects

#### Gut *tert* rescues tissue degeneration

**Fig. 4:**
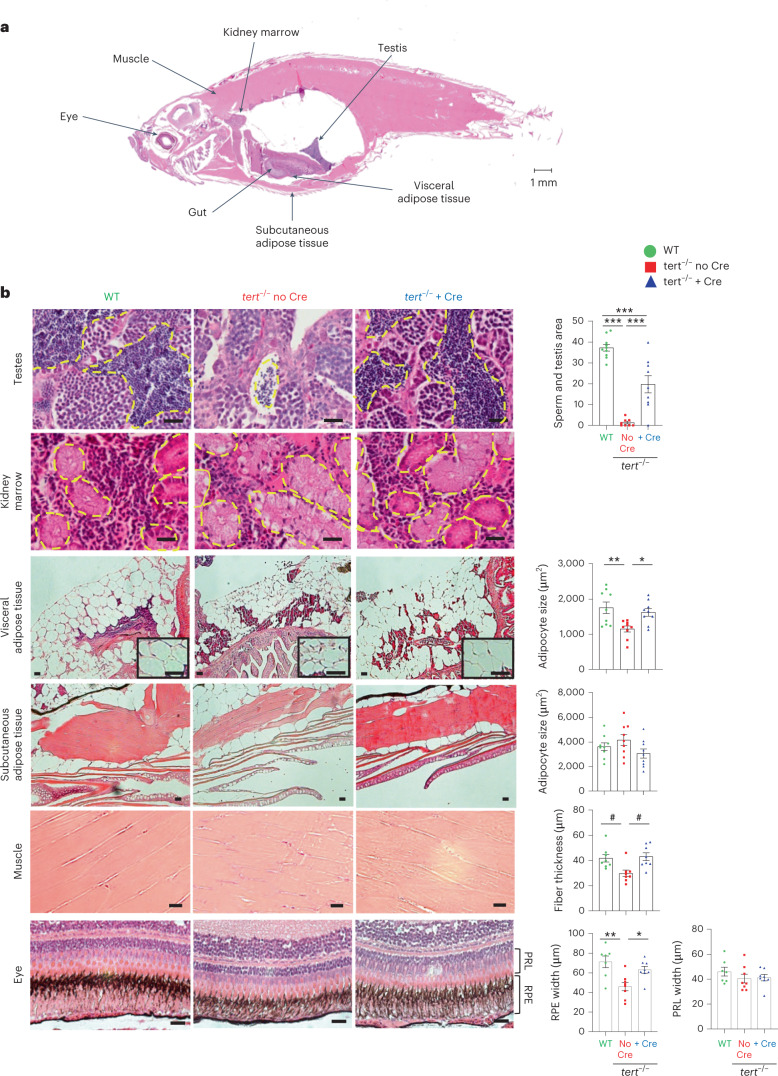
Gut-specific *tert* expression rescues systemic tissue degeneration. Expression of telomerase in the gut of *tert* mutant fish rescued tissue degeneration in the testes, visceral adipose tissue, muscle and eye. **a**, Representative image of a longitudinal section of a zebrafish stained with H&E. The locations of each tissue analyzed in the study are indicated by arrows. **b**, Representative images of testes, kidney, visceral adipose tissue, subcutaneous adipose tissue, muscle and eye from 9-month-old fish stained with H&E (right). Except for the kidney, histological quantifications were performed for each tissue (left), namely the mature spermatids area (*n*_WT_ = 10 fish, $n_{tert^{-/-}\, {\mathrm{no}}\, {\mathrm{Cre}}}$ = 8 fish and $n_{tert^{-/-}\, +\, {\mathrm{Cre}}}$ = 9 fish), adipocyte area (*n*_WT_ = 9 fish, $n_{tert^{-/-}\, {\mathrm{no}}\, {\mathrm{Cre}}}$ = 9 fish and $n_{tert^{-/-}\, +\, {\mathrm{Cre}}}$ = 9 fish), muscle fiber thickness (*n* = 8 fish per group), retinal pigmented epithelium (RPE) and photoreceptor layer (PRL) (*n*_WT_ = 7 fish, $n_{tert^{-/-}\, {\mathrm{no}}\, {\mathrm{Cre}}}$ = 8 fish and $n_{tert^{-/-}\, +\, {\mathrm{Cre}}}$ = 8 fish), respectively. Scale bar, 20 µm. All data are presented as the mean ± s.e.m.; *P < 0.05; **P < 0.01, ***P < 0.001, using a one-way ANOVA and post hoc Tukey test; *P < 0.05, using a Kruskal–Wallis test and post hoc Dunn test. Source data

Intestinal dysfunction is a major feature of aging^4^. To investigate whether gut aging influences overall organismal aging, we explored the systemic impact of gut-specific telomerase expression using histological analyses of a broad spectrum of tissues. As reported previously^14,16,17^, we observed a reduction in mature spermatids area (with severe testes atrophy), in adipocyte size in visceral adipose tissues, in muscle fiber thickness and in retinal pigmented epithelium width in the *tert*^−/−^ no Cre fish compared to WT (Fig. 4a,b). Strikingly, gut-specific telomerase expression recovered all these morphological defects. Of note, no histological differences were detected in kidney marrow between each condition. Moreover, unlike previous observations^14,16^, the adipocyte size of subcutaneous adipose tissue and the photoreceptor layer did not differ between the three genotypes. Therefore, our results indicate that counteracting telomere shortening in the gut systemically ameliorates age-dependant tissue degeneration.

#### Gut short telomeres drive systemic DNA damage and inflammation

**Fig. 5:**
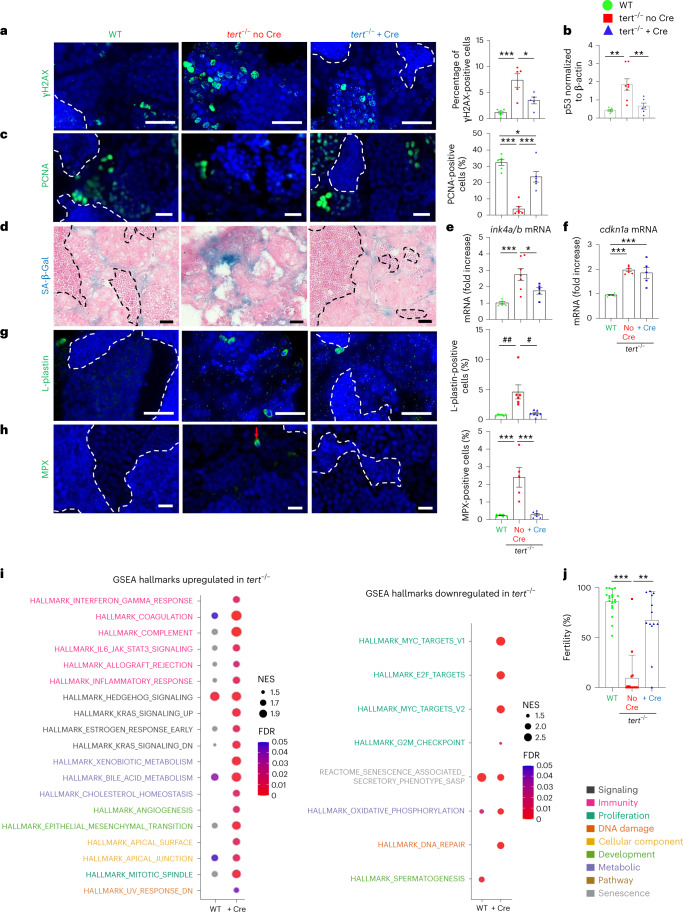
Gut-specific *tert* expression rescues the aging phenotypes of testes. **a**–**e**, Delaying gut aging in *tert*^−/−^ + Cre fish rescues DNA damage, proliferation and senescence in the testes compared to *tert*^−/^^*−*^ no Cre fish. **a**, Representative immunofluorescence images of DNA damage staining (γH2AX, left) and quantification (right, *n*_WT_ = 6, $n_{tert^{-/-}\, {\mathrm{no}}\, {\mathrm{Cre}}}$ = 5 and $n_{tert^{-/-}\, +\, {\mathrm{Cre}}}$ = 5 fish) in the tissue of testes. **b**, Quantification of p53 protein levels (normalized by β-actin) in 9-month-old testes extracts analyzed by western blot (*n*_WT_ = 6, $n_{tert^{-/-}\, {\mathrm{no}}\, {\mathrm{Cre}}}$ = 8 and $n_{tert^{-/-}\, +\, {\mathrm{Cre}}}$ = 8 fish). **c**, Representative immunofluorescence images of proliferation staining (left, PCNA) and quantification (right, *n* = 6 fish per group) in the tissue of testes. **d**, Representative image of SA-β-Gal staining of 9-month-old testes cryosections. **e**,**f**, RT–qPCR analysis of the senescence-associated genes *ink4a/b* (p15/16) (**e**) and *cdkn1a* (p21) (**f**) expression in testes samples (*n*_WT_ = 6 and 5, $n_{tert^{-/-}\, {\mathrm{no}}\, {\mathrm{Cre}}}$ = 7 and 6 and $n_{tert^{-/-}\, +\, {\mathrm{Cre}}}$ = 5 and 5 fish, respectively). **g**, Representative immunofluorescence images of immune cell staining (left, L-plastin) and quantification (right, *n*_WT_ = 6, $n_{tert^{-/-}\, {\mathrm{no}}\, {\mathrm{Cre}}}$ = 6 and $n_{tert^{-/-}\, +\, {\mathrm{Cre}}}$ = 7 fish) in testes tissues. **h**, Representative immunofluorescence images of neutrophil staining (left, MPX) and quantification (right, *n*_WT_ = 6, $n_{tert^{-/-}\, {\mathrm{no}}\, {\mathrm{Cre}}}$ = 5 and $n_{tert^{-/-}\, +\, {\mathrm{Cre}}}$ = 6 fish) in the tissue of testes. **i**, Identification of upregulated (left) or downregulated (right) hallmarks in the testes of *tert*^−/−^ no Cre fish compared to either WT or *tert*^−/−^ + Cre, based on GSEA. The NES depict to what degree the pathway’s genes are overrepresented in WT or *tert*^−/−^ + Cre, compared to *tert*^−/−^ no Cre fish. **j**, Quantification of male fertility of fish determined by counting the percentage of fertilized eggs (detected by successful embryogenesis events) after individually crossing 9-month-old males with a young (3–6-month-old) WT female (*n*_WT_ = 19, $n_{tert^{-/-}\, {\mathrm{no}}\, {\mathrm{Cre}}}$ = 16 and $n_{tert^{-/-}\, +\, {\mathrm{Cre}}}$ = 13 fish). All analyses were done on sections of 9-month-old fish testes or extracts. Scale bar, 20 µm. The dashed lines delineate the area of mature spermatids. All data are presented as the mean ± s.e.m. *P < 0.05, **P < 0.01, ***P < 0.001, using a one-way ANOVA and post hoc Tukey test; and *P < 0.05, **P  < 0.01 using a Kruskal–Wallis and post hoc Dunn test. The RT–qPCR graphs represent the mean ± s.e.m. Note the mRNA fold increase after normalization by *rps11* gene expression levels. Source data

**Fig. 6:**
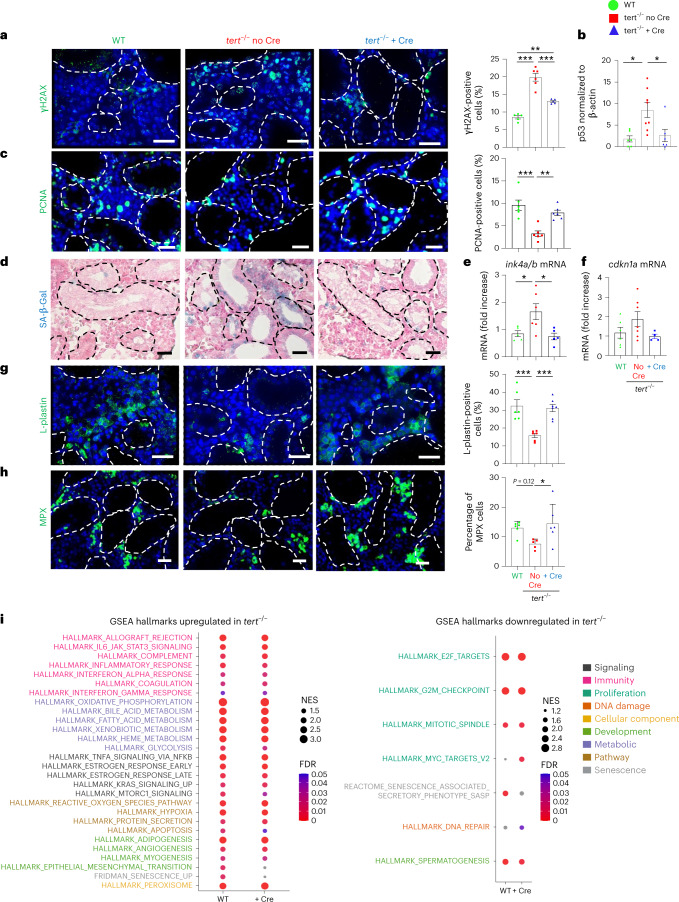
Gut-specific *tert* expression rescues aging of the hematopoietic system (kidney marrow). **a**–**f**, Delaying gut aging in *tert*^−/−^ + Cre fish rescues DNA damage, proliferation and senescence in the kidney marrow when compared to *tert*^−/−^ no Cre fish. **a**, Representative immunofluorescence images of DNA damage staining (left, γH2AX) and quantification (right, *n*_WT_ = 5, $n_{tert^{-/-}\, {\mathrm{no}}\, {\mathrm{Cre}}}$ = 6 and $n_{tert^{-/-}\, +\, {\mathrm{Cre}}}$ = 5 fish) in 9-month-old kidney marrow tissues. **b**, Quantification of p53 protein levels in 9-month-old kidney extracts analyzed by western blot (*n*_WT_ = 6, $n_{tert^{-/-}\, {\mathrm{no}}\, {\mathrm{Cre}}}$ = 8 and $n_{tert^{-/-}\, +\, {\mathrm{Cre}}}$ = 6 fish). **c**, Representative immunofluorescence images of proliferation staining (left, PCNA) and quantification (right, *n* = 6 fish per group) in 9-month-old kidney marrow tissues. **d**, Representative images of SA-β-Gal staining of 9-month-old kidney marrow cryosections. **e**,**f**, RT–qPCR analysis of senescence-associated genes *ink4a/b* (*p15/16*) (*n*_WT_ = 5, $n_{tert^{-/-}\, {\mathrm{no}}\, {\mathrm{Cre}}}$ = 6 and $n_{tert^{-/-}\, +\, {\mathrm{Cre}}}$ = 5 fish) (**e**) and *cdkn1a* (p21) (*n*_WT_ = 6, $n_{tert^{-/-}\, {\mathrm{no}}\, {\mathrm{Cre}}}$ = 7 and $n_{tert^{-/-}\, +\, {\mathrm{Cre}}}$ = 4 fish) (**f**) expression in 9-month-old kidney marrow samples. **g**–**i**, *tert* mRNA expression in the gut of *tert*^−/−^ fish (*tert*^−/−^ + Cre fish) have beneficial hematopoietic effects by reducing kidney marrow inflammation and increasing immune compartment compared to *tert*^−/−^ no Cre fish. **g**, Representative immunofluorescence images of immune cell staining (left, L-plastin) and quantification (right, *n*_WT_ = 6, $n_{tert^{-/-}\, {\mathrm{no}}\, {\mathrm{Cre}}}$ = 6 and $n_{tert^{-/-}\, +\, {\mathrm{Cre}}}$ = 7 fish) in the tissue of 9-month-old testes. **h**, Representative immunofluorescence images of neutrophil staining (left, MPX) and quantification (right, *n*_WT_ = 6, $n_{tert^{-/-}\, {\mathrm{no}}\, {\mathrm{Cre}}}$ = 5 and $n_{tert^{-/-}\, +\, {\mathrm{Cre}}}$ = 6 fish) in the tissue of 9-month-old kidney marrow. **i**, Identification of upregulated (left) or downregulated (right) hallmarks in the kidney marrow of *tert*^−/−^ no Cre compared to either WT or *tert*^−/−^ + Cre fish based on GSEA. The NES depicts to what degree the pathway genes are overrepresented in WT or *tert*^−/−^ + Cre, compared to *tert*^−/−^ no Cre fish. Scale bar, 20 µm. The dashed lines delineate the kidney tubules. All data are presented as the mean ± s.e.m. (*P < 0.05; **P < 0.01, ***P < 0.001, using a one-way ANOVA and post hoc Tukey test). The western blot graphs represent the mean ± s.e.m. of p53 normalized by β-actin band intensities. All RT–qPCR graphs represent the mean ± s.e.m. mRNA fold increase after normalization by *rps11* gene expression levels. Source data

We decided to study the extent of systemic aging recovery of specific distant organs. Given the importance of anemia in patients with TBD^42,43^, and the drastic histological phenotype seen in the testes of *tert*^−/−^ no Cre fish, we further detailed the rescue in the kidney marrow (the adult hematopoietic organ in zebrafish) and testes (the reproductive system). As described previously^14,17^, we observed increased γH2AX-positive cells and high levels of p53 in both the testes and kidney marrow of *tert*^−/−^ no Cre fish (Figs. 5a,b and 6a,b). These organs were affected by reduced cell proliferation and high senescence (Figs. 5c–f and 6c–f). In the kidney, even though most cells affected by DNA damage and low proliferation reside in the hematopoietic compartment, we also observed SA-β-Gal-positive cells in kidney tubules, suggesting that both hematopoietic and nephrotic functions were affected in *tert*^−/−^ no Cre fish.

Surprisingly, gut-specific telomerase expression in *tert*^−/−^ mutants resulted in a reduction in DNA damage, p53 levels and recovery of cell proliferation in both testes and kidney marrow (Figs. 5a–c and 6a–c). Moreover, SA-β-Gal and *ink4a/b* (p15/16) mRNA levels were reduced to WT levels in *tert*^−/−^ + Cre testes and kidney marrow (Figs. 5d,e and 6d,e). While *cdkn1a* (p21) mRNA levels were maintained in the testes of *tert*^−/−^ no Cre fish, these were rescued in kidney marrow of *tert*^−/−^ + Cre fish (Figs. 5f and 6f). Consistent with what we observed in the gut, apoptosis did not vary in either testes or kidney marrow (Extended Data Fig. 2b,c). Therefore, gut-specific telomerase expression unexpectedly rescues DNA damage, proliferation and senescence in both the reproductive and hematopoietic systems.

The increased immune infiltrates present in the testes of *tert*^−/−^ no Cre fish were also reverted in the *tert*^−/−^ + Cre fish (Fig. 5g,h). However, in contrast to the gut and testes, we observed a considerable reduction of immune cells in the kidney marrow of *tert*^−/−^ no Cre fish (Fig. 6g,h). These numbers were reverted to WT levels in *tert*^−/−^ + Cre fish. Thus, our results provide evidence for a decreased reserve pool of immune cells in *tert*^−/−^ no Cre fish that is rescued by gut-specific telomerase expression. Decline of immune cells in the kidney marrow constitutes an early sign of hematopoietic dysfunction, which is comparable to the bone marrow failure described in patients with TBD^42,43^.

**Extended Data Fig. 4:**
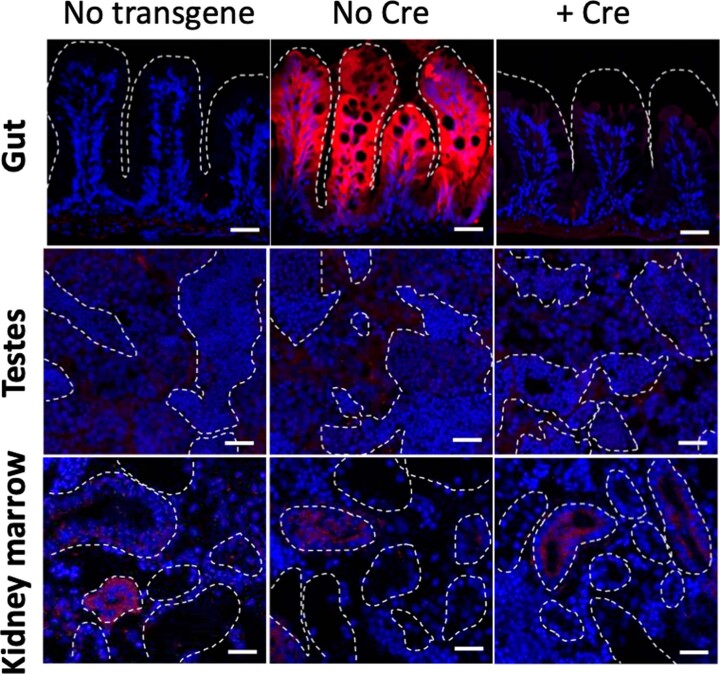
The *fabp2* promoter regulates gut specific transgene expression. Representative images of DsRed immunofluorescence staining of cryosection from gut, testes and kidney marrow cryosections from zebrafish containing no transgene, No Cre *fabp2: loxp-dsred-loxp-tert-t2a-cfp transgene* or Cre-induced *fabp2*: *loxp-dsred-loxp-tert-t2a-cfp transgene*.

**Extended Data Fig. 5:**
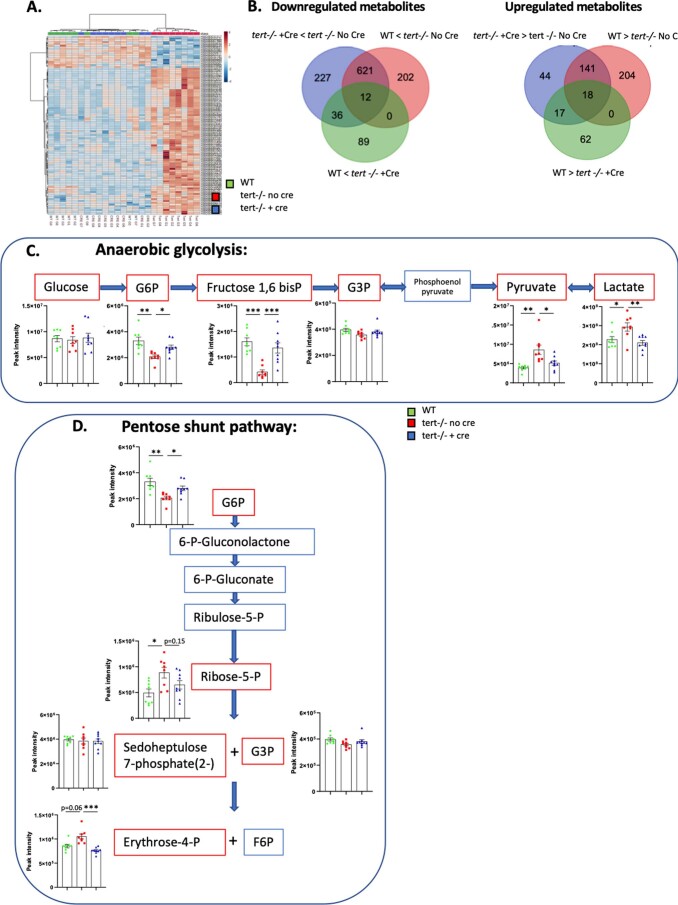
Gut-specific telomerase activity rescues gut metabolomic profile. **a**. Heatmap clustering analysis based on untargeted metabolomic data of 9-month-old gut samples. A clustering between *tert*^−/−^ +Cre and WT while *tert*^−/−^ No Cre group was clearly distinguishable from others. **b**. Venn diagram representing downregulated (left panel) or upregulated (right panel) gut metabolites comparing the three conditions. Most metabolites detected in the gut of 9 months-old fish are concomitantly down or up-regulated in *tert*^−/−^ +Cre and WT groups compared to *tert*^−/−^ No Cre fish. **c, d**. Anaerobic glycolysis and pentose shunt metabolic profiles are rescued to WT levels in the gut of *tert*^−/−^ +Cre compared *tert*^−/−^ No Cre fish. Metabolomic analysis of the anaerobic glycolysis (**C**.) and pentose shunt pathways (**D**.) in gut of 9-month-old fish. All data are represented as mean +/− SEM (N_WT_ = 8 fish, N_tert−/− No Cre_ = 8 fish and N_tert−/− +Cre_ = 9 fish; * p-value<0.05; ** p-value<0.01, *** p-value<0.001, using one-way ANOVA and post-hoc Tukey tests). Red squares: detected metabolites; blue squares: undetected metabolites. Source data

**Extended Data Fig. 6:**
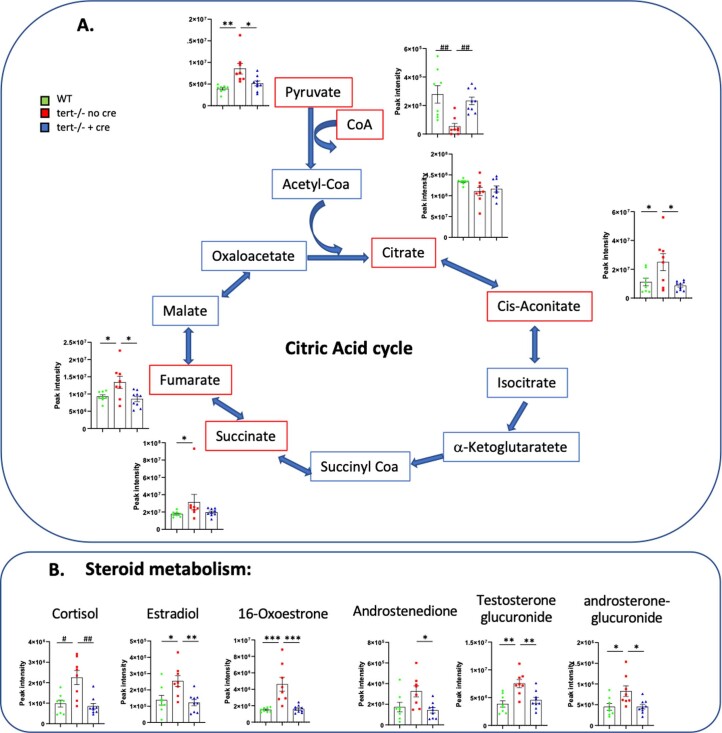
Gut-specific telomerase activity rescues citric acid cycle and steroid metabolism alterations in the gut of tert^−/−^ fish. **a**, **b**. Metabolomic analysis of the citric acid cycle (**A**.) and steroid metabolism (**B**.) in gut of 9-month-old fish. Citric cycle and steroid metabolic profiles in the gut of *tert*^−/−^ +Cre is similar to WT when compared *tert*^−/−^ No Cre fish. All data are represented as mean +/- SEM (N_WT_ = 8 fish, N_tert−/− No Cre_ = 8 fish and N_tert−/− +Cre_ = 9 fish; * p-value<0.05; ** p-value<0.01, using one-way ANOVA and post-hoc Tukey tests; ## p-value<0.01, using Kruskal-Wallis and post-hoc Dunn’s tests). Red squares: detected metabolites; blue squares: undetected metabolites. Source data

**Extended Data Fig. 7:**
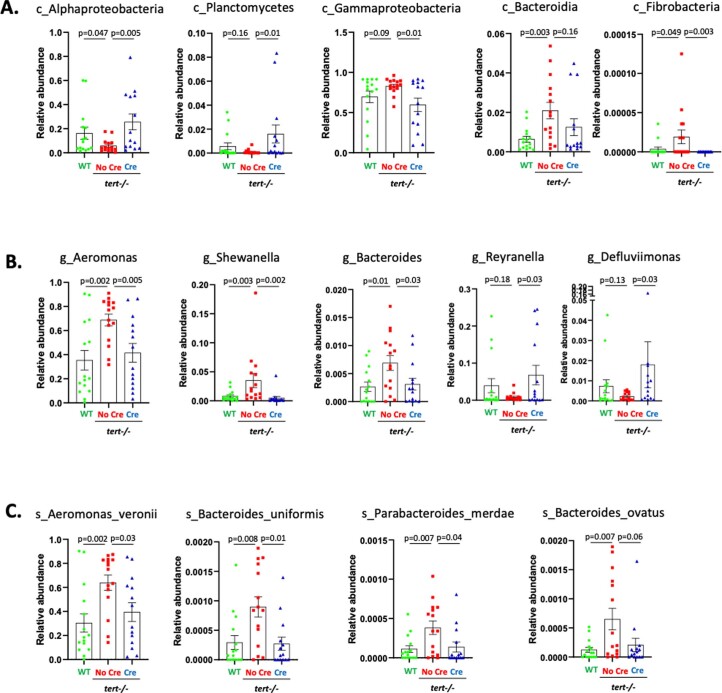
Gut-specific tert expression rescues alterations of gut microbiota composition. **a**–**c**. *tert* mRNA expression in gut of *tert*^−/−^ fish (*tert*^−/−^ +Cre) recapitulates bacteria abundance at the class and species levels to WT profile compared to *tert*^−/−^ No Cre in which pathogenic bacteria are enriched. Relative abundance analysis of bacteria at the level of class **(A.)**; genus **(B.)** and species **(C.)** N_WT_ = 15 fish, N_tert−/− No Cre_ = 15 fish and N_tert−/− +Cre_ = 14 fish; p values were determined using two-sided Multiple hypothesis-test for sparsely sampled features and false discovery rate (FDR). Source data

To ensure that these effects were not due to leaky *fabp2* enterocyte promoter expression in other tissues, we performed quantitative PCR with reverse transcription (RT–qPCR) experiments on testes and kidney marrow. While a clear induction of the *tert* transgene and total *tert* mRNA was observed in the gut of *tert*^−/−^ + Cre fish, no expression of the transgene was detected in either distant organ (Extended Data Fig. 1a,b). Consistently, the DsRed reporter for transgene expression showed that the *fabp2* promoter was solely expressed in gut differentiated cells but not the testes or kidney marrow (Extended Data Fig. 4). As expected, we observed neither telomerase activity nor telomere elongation in distant organs (Extended Data Fig. 1g–p). In contrast, telomere shortening was observed in *tert*^−/−^ + Cre kidney marrow and testes, similar to the telomere length of *tert*^−/−^ no Cre fish. These experiments support a systemic role of gut-specific telomerase expression.

Fertility decreases during natural aging of zebrafish and most mammals. Loss of male fertility is accelerated in murine and fish premature *tert*^−/−^ aging models^14,44^. To test the male reproductive function, we crossed 9-month-old males of the three groups with young WT females. The percentages of fertilized eggs spawned by young females were scored as a male fertility index. Consistent with a reduction of mature spermatid content, *tert*^−/−^ no Cre male fish exhibited a drastic reduction of fertility (Fig. 5j). In contrast, we observed a full recovery of male fertility in *tert*^−/−^ + Cre fish. Therefore, gut-specific telomerase expression not only improves cellular and morphological defects of the male reproductive system, but also rescues age-dependent loss of fertility.

Finally, to understand the mechanism through which gut decline influences aging of distant organs, we analyzed the transcriptomics profile of testes and kidney marrow. As in the gut, we observed similar GSEA hallmark profiles when comparing *tert*^−/−^ no Cre to either WT or *tert*^−/−^ + Cre (Figs. 5i and 6i), indicating that telomerase expression in the gut rescues the transcriptomics profile of *tert* mutant testes and kidney marrow. As in the gut, we observed a marked enrichment of hallmarks of inflammation and a reduction of proliferation-related genes in *tert*^−/−^ no Cre. Hallmarks of metabolic pathways were also upregulated in *tert*^−/−^ no Cre indicating a metabolic shift in this condition. Unexpectedly, even though senescence was higher in all organs of *tert*^−/−^ no Cre fish (Figs. 5d–f and 6d–f), in contrast to the gut, we observed a downregulation of the SASP hallmark in both the testes and kidney marrow. This result suggests that paracrine senescence of distant organs initiated by the gut may have limited expression of SASP molecules, as previously observed in secondary senescent cells^45^.

#### Gut *tert* extends *tert*^−/−^ lifespan and improves WT healthspan

**Fig. 7:**
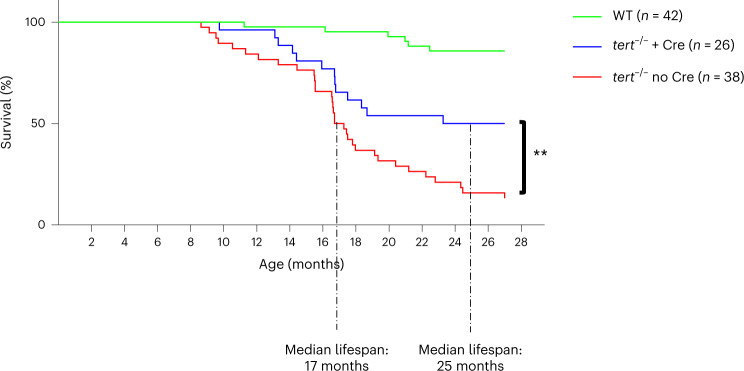
Gut-specific *tert* expression extends the lifespan of *tert*^−/−^ zebrafish. Gut-specific telomerase activity extends the lifespan, increasing median life from 17 months in *tert*^−/−^ no Cre fish to 24 months in *tert*^−/−^ + Cre fish. The survival curve of WT (*n* = 42 fish), *tert*^−/−^ no Cre (*n* = 38 fish) and *tert*^−/−^ + Cre (*n* = 26 fish) zebrafish (**P < 0.01 using the log-rank test) is shown. Source data

We next tested whether telomerase expression in the gut would influence the lifespan of zebrafish. As described previously^14–16^, telomere shortening in *tert*^−/−^ no Cre fish reduces the average lifespan to 12–18 months compared to more than 42 months in WT fish (Fig. 7). Strikingly, delaying gut aging was sufficient to extend the average lifespan of *tert*^−/−^ fish by approximately 40%. The average lifespan of *tert*^−/−^ no Cre fish was extended from 17 months to 24 months in *tert*^−/−^ + Cre fish (Fig. 7). Nevertheless, telomerase expression in the gut was not sufficient to fully rescue life expectancy to WT levels, suggesting that telomere shortening in other organs may be limiting in later stages.

**Fig. 8:**
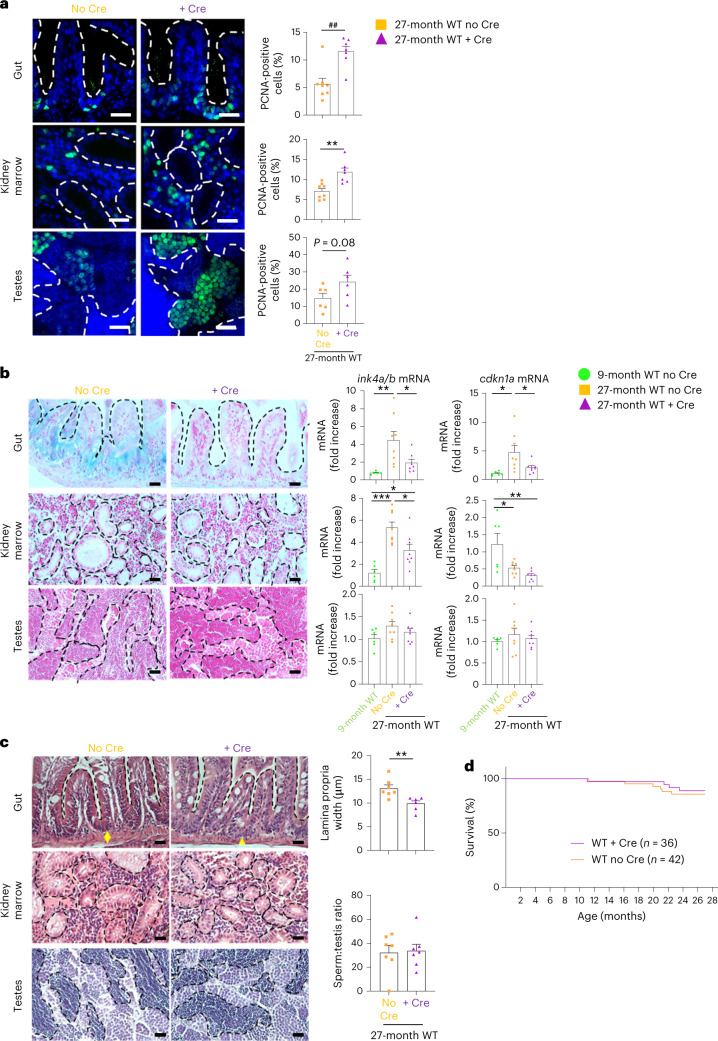
Gut-specific *tert* expression extends the healthspan of naturally aged zebrafish. Expression of *tert* transgene in the gut of WT fish delays local aging phenotypes such as proliferation, senescence and tissue degeneration. This leads to beneficial systemic impact improving early aging phenotypes such as proliferation capacity. **a**, Representative immunofluorescence images of proliferation staining (left, PCNA) and quantification (right) in the gut, testes and kidney marrow of 27-month-old WT zebrafish expressing (WT + Cre; *n* = 7 fish for the gut and kidney marrow and *n* = 6 fish for the testes) or not expressing (WT no Cre; *n* = 8 fish for the gut and kidney marrow and *n* = 6 fish for the testes) *tert* transgene in the gut. **b**, Representative images of SA-β-Gal staining of gut, testes and kidney marrow sections of 24-month-old WT zebrafish expressing (WT + Cre) or not expressing (WT no Cre) the *tert* transgene in the gut (left). RT–qPCR analysis of senescence-associated genes *ink4a/b* (p15/16) and *cdkn1a* (p21) in the gut, testes and kidney marrow of either 9- (*n* = 6 fish) or 27-month-old WT zebrafish expressing (WT + Cre; *n* = 8 fish) or not expressing (WT no Cre; *n* = 8 fish) *tert* mRNA in the gut (right). **c**, Representative H&E-stained sections of the gut, testes and kidney marrow of 27-month-old WT zebrafish expressing (WT + Cre) or not expressing (WT no Cre) the *tert* transgene in the gut (left) and respective quantifications of the width of the gut lamina propria (right; *n* = 7 fish for WT no Cre and *n* = 6 fish for WT + Cre) and mature spermatid area (right; *n* = 7 fish for WT no Cre and WT + Cre). The yellow arrows indicate the width of the lamina propria quantified on the left. The dashed lines delineate the mature area of the spermatids. **d**, Survival curve of WT no Cre (*n* = 42 fish; similar to the WT curve in Fig. 7) and WT + Cre (*n* = 36 fish) zebrafish. All data are presented as the mean ± s.e.m. *P < 0.05, **P < 0.01, ***P < 0.001, using a two-tailed unpaired *t*-test for **a**,**c** or a one-way ANOVA and post hoc Tukey tests for **b**; **P < 0.01, a using two-tailed Mann–Whitney *U*-test). All RT–qPCR graphs represent the mean ± s.e.m. mRNA fold increase after normalization by *rps11* gene expression levels. Scale bar, 20 µm. Source data

Finally, to extend our discovery to the natural aging of zebrafish, we studied the recovery of 24–27-month-old WT zebrafish expressing (WT + Cre) or not expressing (WT no Cre) the *tert* transgene in the gut. At that age, we did not yet distinguish differences in survival between the two groups (Fig. 8d). However, we observed that gut-specific telomerase expression in WT fish increased cell proliferation, reduced gut lamina propria width and counteracted cell senescence in the gut compared to WT no Cre (Fig. 8a–c). As observed in *tert*^−/−^ fish, expressing telomerase in the gut of WT fish is sufficient to improve the proliferative capacity of distant organs such as the testes and kidney marrow (Fig. 8a). Except for a partial rescue of *ink4a/b* (p15/16) mRNA levels in kidney marrow, we did not observed signs of senescence in either distant organ using SA-β-Gal assays or assessing for transcription levels of *ink4a/b* (p15/16) and *cdkn1a* (p21) (Fig. 8b). Consistently, we did not observe histological defects in distant organs (Fig. 8c). Therefore, while 24–27-month-old WT fish do not fully exhibit natural aging phenotypes, our data revealed that delaying gut aging by gut-specific *tert* overexpression is sufficient to counteract the early signs of aging, such as loss of proliferative capacity. It also confirms that the gut is one of the earliest organs affected in natural aging.

## Discussion

The gut is a central organ in aging and it constitutes the most extensive and selective living barrier to the external environment. Besides its function in nutrient uptake, it has an important role in immune modulation and supports a complex interaction with the gut microbiota^4^.

Broader keratinocyte promoter-driven telomerase expression was shown to counteract degenerative phenotypes of late-generation *tert*^−/−^ mice^46,47^. Aging phenotypes were ameliorated, not only in the gut, but also in other organs such as the testes, kidney and skin. However, in these studies, *tert* expression was not targeted to a specific organ. The dePinho laboratory recently showed that telomere shortening in mice triggers gut inflammation through the YAP pathway^48^. Mosaic expression of *tert* in the LGR5 cells of *tert*^−/−^ mice improved intestinal function and inflammation. However, no significant systemic effects were reported apart from body weight gain and a modest increase in survival. Consistently, we showed that YAP target genes were likewise induced in *tert*^−/−^ no Cre fish. These were rescued in *tert*^−/−^ + Cre fish that not only reverted the YAP pathway, but also rescued local inflammation. Moreover, we showed that counteracting gut telomere dysfunction also delays remote organ dysfunction and overall organismal aging.

In our study, we showed that enterocyte-specific telomerase expression in zebrafish is sufficient to prolong maintenance of gut homeostasis with age. Rescue of gut aging was observed in the context of a minor, but significant, telomere elongation in the gut of *tert*^−/−^ fish. Consistent with an increase in telomere length of the shortest telomere population (tenth percentile), this was sufficient to abrogate DNA damage, higher p53 levels and cell senescence in this organ. Nevertheless, considering the potential noncanonical roles of telomerase in proliferation and resistance to oxidative stress^49^, we cannot exclude these effects in our work. Similarly, trace amounts of *fabp2* transcripts were previously reported in zebrafish in the liver, brain and kidney marrow, but not in the testes^50^. While we did not measure any *fabp2*-dependent transgene mRNA in other tissues, we cannot exclude that undetected spurious expression may participate in the systemic effects.

Common laboratory mice possess long telomeres (ranging from 40 to 150 kb) compared to humans and zebrafish (5–15 kb). Therefore, producing telomerase-deficient mice requires several generations of in-breeding (G3–G4) before mice show premature aging phenotypes^44,51^. We designed a new vertebrate model to study the systemic effects of delaying aging of an individual organ, the gut, by maintaining telomere length through enforced telomerase expression. We report that delaying telomere-dependent gut aging has beneficial systemic effects not only in a premature aging model, that is, *tert*^−/−^ mutants, but also in a context of natural aging of zebrafish. Notably, our study indicates that proliferative organs, such as the reproductive or hematopoietic systems, can conserve their regenerative capacity even in a context of shorter telomeres. This was observed in the rescue of telomerase deficiency by *tp53* mutations in mice and zebrafish^15,52^. Thus, maintenance of proliferative capacity and tissue integrity in these organs relies on external cues from an aging gut. We propose that the intestine is at the top of a cascade of events that initiate systemic aging; thus, restoring intestine integrity can result in organismal rejuvenation.

How would gut aging influence the entire organism? Aging is associated with persistent DNA damage and inflammation^2^. In recent years, we have seen a flurry of studies supporting the role of inflammation and SASP in inducing paracrine senescence in remote tissues^53,54^. Senescent cells accumulate with age in tissues and promote aging by secreting molecules such as inflammatory cytokines, chemokines and other molecules, also known as SASP^54^. Clearance of these cells delays age-associated defects and leads to lifespan extension^5,6^. We previously reported that some organs in zebrafish, such as the kidney marrow, exhibit cellular senescence before exhibiting critically short telomeres^14,15^. We now show that gut of *tert*^−/−^ no Cre fish accumulates senescent cells and expresses SASP and inflammatory molecules, such as interleukin-6 (IL-6) or transforming growth factor-β (TGFβ). Senescent cells can induce ROS-mediated DNA damage in distant tissues by secreting TGFβ and IL-1β^55,56^. Therefore, we anticipate that inflammation and SASP factors secreted by an aging gut trigger DNA damage in distant organs. This mechanism results in secondary senescence, affecting cell proliferation systemically and leading to loss of tissue homeostasis and aging in the entire organism.

Alterations in gut microbiota have been linked to aging^34,57^ and are involved in age-related systemic inflammation^31^. Specific bacterial taxa are capable of inducing gut cell senescence but also in distant organs (for example, the liver)^58,59^. We show that delaying gut aging counteracted gut microbiota dysbiosis. We anticipate that, due to limiting gut telomere length, increasingly dysbiotic microbiota will cause systemic aging, either directly through microbial components or by triggering systemic inflammation or senescence. This idea is supported by work showing that stool transfers from young to middle-aged individuals is sufficient to extend the lifespan of short-lived killifish^60^.

We noticed an accumulation of methionine and its metabolites (*S*-adenosylmethionine (SAM), *S*-adenosylhomocysteine and homocysteine) in the gut of *tert*^−/−^ no Cre fish. Similar enrichment with age has been reported in humans and mice^29,61^. Dietary methionine restriction or impeding SAM accumulation extends the lifespan in different animal models^4,62–66^. Hyperhomocysteinemia has also been implicated in several age-related disorders^61^. Mechanistically, deleterious effects of methionine and its metabolites involves DNA methylation drift, mTOR activation, inflammation and oxidative stress^4,30,63^. We suggest that propagation of these molecules throughout the zebrafish organism contributes to systemic aging.

Overall, the present work describes a central role of telomere shortening in the gut during the aging of a vertebrate organism. We provide several mechanistic clues on how this organ influences aging of the entire organism, namely through microbiota dysbiosis, inflammation and SASP, and dysregulation of methionine metabolism. Our future work will disentangle these mechanisms by targeting them independently in a unique organ, the gut, as an exciting strategy to extend the healthspan and lifespan.

## Methods

### Plasmid construct

Zebrafish tert cDNA was obtained using the TertFL-pCR-II-Topo plasmid provided by the Kishi laboratory^58^. Using Gibson assembly recombination methods, *tert* cDNA and enhanced constitutively fluorescent protein (eCFP) cDNA were linked by the *T2A* sequence and inserted into the *Ubi: loxP-dsRed-loxP-EGFP* vector plasmid (a gift from the Zon laboratory derived from *Ubi: Switch* and *lmo2: Switch* contructs^59^). The enterocyte-specific intestinal fatty acid binding protein promoter (−2.3 kb *fabp2*, also called *i-fabp*) was amplified using high-fidelity PCR (iProof High-Fidelity DNA Polymerase; Bio-Rad Laboratories) from the *p5E–2.3ifabp* plasmid (gifted by the Rawls laboratory). The −2.3-kb *fabp2* PCR product was then cloned into the *Ubi: loxP-dsRed-loxP-tert-T2A-CFP* using sfI/FseI digestion to provide the final construct*: fabp2*: *loxP-dsRed-loxP-tert-T2A-CFP*.

### Generation of transgenic fish

*Tol2* mRNA was synthesized with SP6 RNA polymerase from the pCS2FA-transposase plasmid (Tol2Kit) using the mMESSAGE mMACHINE SP6 transcription kit (Invitrogen). One-cell-stage zebrafish embryos were microinjected with 1.4 nl of a mixture containing 25 ng µl^−1^ of linearized plasmid and 100 ng µl^−1^ of *Tol2* mRNA, diluted with RNase-free water. Injected fish were raised to adulthood and germline-transmitting fish were selected and outcrossed to WT AB until a single-copy transgenic line Tg was obtained (*fabp2: loxP-dsRed-loxP-tert-T2A-CFP*).

### Zebrafish lines and maintenance

Zebrafish were maintained in accordance with institutional and national animal care protocols. Generation and maintenance of the telomerase mutant line *tert* AB/hu3430 (referred in this work as *tert*^+/−^) were described previously^14,15,17^. This line was outcrossed with Tg(*fabp2: loxP-dsRed-loxP-tert-T2A-CFP*) line to obtain a stock that combined both transgenics. All stocks were kept in heterozygous form for the *tert* mutation and were strictly maintained by outcrossing to AB strains to avoid haploinsufficiency effects in the progeny.

Experimental fish were obtained by crossing *tert*^+/−^ fish with *tert* ^+/−^*; fabp2: loxP-dsRed-loxP-tert-T2A-CFP*. Their embryos were microinjected with 1.4 nl of either 25 ng µl^−1^ Cre mRNA diluted in RNase-free water (Cre-induced fish) or RNase-free water alone (mock-injected fish). This experimental setup provided sibling fish that were either *tert*^−/−^; *fabp2: loxP-dsRed-loxP-tert-T2A-CFP* (mock-injected *tert*^−/−^, referred to as *tert*^−/−^ no Cre), *tert*^−/−^*; fabp2*: *tert-T2A-CFP* (Cre-induced *tert*^−/−^, referred to as *tert*^−/−^ + Cre) or *tert*^+/+^*; fabp2: loxP-dsRed-loxP-tert-T2A-CFP* (mock-injected WT, referred to as WT). Overall characterization of these three genotypes was performed in F1 siblings at 9 months of age. Due to a male sex bias in our crosses, primarily observed in the *tert*^−/−^ progeny, we were unable to obtain significant numbers of females for analysis; thus, all but our survival data are restricted to males.

### Fertility assays

To assess male fertility, 9-month-old males from the three different genotypes were housed separately overnight in external breeding tanks with a young 3–6-month-old WT female. Breeding pairs were left to cross and lay eggs the following morning. Embryos were collected approximately 2 h after fertilization and allowed to develop at 28 °C. Assessment of egg fertilization and embryo viability was conducted between 2 and 4 h after fertilization. At least 14 independent crosses were conducted for each genotype to evaluate male fertility. Only successful breeding trials were scored. Events where females laid a normal clutch of eggs were scored.

### Histology

Zebrafish were killed by lethal dose of 1 g l^−1^ of MS-222 (Sigma-Aldrich), fixed for 72 h in 10% neutral buffered formalin and decalcified in 0.5 M EDTA for 48 h at room temperature. Whole fish were paraffin-embedded to create 5-µm sagittal section slides. Slides were stained with H&E for histopathological analysis. Microphotographs (*n* ≥ 6 fish per genotype) were acquired with a Leica DM4000 B microscope coupled to a Leica DFC425 C camera (Leica Microsystems).

### Senescence-associated β-galactosidase staining

Tissues were fixed with 4% paraformaldehyde for 3 h at 4 °C. After washing with PBS, they were incubated in 30% sucrose (Sigma-Aldrich) at 4 °C until sinking (24–48 h). Fixed tissues were then embedded in optimal cutting temperature medium (MM France) and kept at −80 °C. Senescence-associated β-galactosidase staining was performed on slides of 5-µm cryosections using the Senescence β-Galactosidase Staining Kit (catalog no. 9860, Cell Signaling Technology) following manufacturer’s instructions. After 16-h (testes, kidney marrow) or 3-h (gut) incubations with the X-Gal staining solution at 37 °C, slides were washed with PBS and counterstained for 1 min with Nuclear Fast Red solution (Sigma-Aldrich) before being dehydrated and mounted.

### Immunofluorescence

Deparaffinized and rehydrated slides were microwaved for 20 min at 550 W in citrate buffer (10 mM sodium citrate, pH 6) to allow for antigen retrieval. Slides were washed twice in PBS for 5 min each and blocked for 1 h at room temperature in 0.5% Triton X-100 and 5% normal goat serum in PBS (blocking solution). Subsequently, slides were incubated overnight at 4 °C with 1:50 dilution of primary antibody in the blocking solution. The following primary antibodies were used: rabbit polyclonal anti-histone H2A.XS139ph (γH2AX, phospho Ser139, 1:50 dilution, catalog no. GTX127342, GeneTex); rabbit polyclonal anti-L-plastin (1:100 dilution, catalog no. GTX124420, GeneTex); mouse monoclonal antibody anti-PCNA (1:50 dilution, catalog no. sc56, Santa Cruz Biotechnology); and rabbit polyclonal anti-MPX (1:50 dilution, catalog no. GTX128379, GeneTex). After two PBS washes, overnight incubation at 4 °C was performed with 1:500 dilution of the Alexa Fluor 488 goat anti-rabbit or anti-mouse secondary antibody (Invitrogen). Finally, after 4,6-diamidino-2-phenylindole staining (DAPI) (Sigma-Aldrich), slides were mounted in DAKO Fluorescence Mounting Medium (Sigma-Aldrich).

Apoptosis was detected using the In Situ Cell Death Detection Kit (Roche) as described previously^14,17^. Briefly, deparaffinized sections were permeabilized by 1-h incubation at 37 °C with 40 μg ml^−1^ proteinase K (Sigma-Aldrich) in 10 mM Tris-HCl, pH 7.4. After washing with PBS, slides were incubated for 1 h at 37 °C with TUNEL Label Mix (according to the manufacturer’s instructions) before DAPI staining and mounting.

Immunofluorescence images were acquired on the Delta Vision Elite microscope (GE Healthcare) using an OLYMPUS ×20/0.75 objective. For quantitative and comparative imaging, equivalent image acquisition parameters were used. The percentage of positive nuclei was determined by counting a total of 500–1,000 cells per slide (*n* ≥ 6 zebrafish per genotype).

### Western blot

Proteins for the western blot were extracted according to the manufacturer’s protocol with TRIzol (Invitrogen) and protein in microliters was quantified with QuantiPro BCA Assay Kit (Sigma-Aldrich). A total of 30 μg of protein was loaded per lane and resolved in 10% resolution gel at 120 V for 2 h and transferred to a nitrocellulose membrane (LI-COR) at 20 V for 70 min with the Trans-Blot SD Semi-Dry Electrophoretic Transfer Cell system (Bio-Rad Laboratories). Transfer and quality were checked with Ponceau staining (VWR) and washed thoroughly with 1× PBS with 0.05% Tween 20 (PBST). Membranes were incubated with primary antibody (anti-p53, 1:1,000 dilution, catalog no. 55342, AnaSpec and anti-actin 1:1,000 dilution, catalog no. A2066, Sigma-Aldrich) overnight at 4 °C with gentle shaking after 1-h blocking in 5% skimmed milk (Sigma-Aldrich) in 0.05% PBST at room temperature with gentle shaking. After three washes with 0.05% PBST, membranes were incubated with secondary antibody (anti-rabbit, 1:10,000, catalog no. sc-2357, Santa Cruz Biotechnology) for 1 h at room temperature with gentle shaking. This was followed by three washes with 0.05% PBST and membranes were then revealed with Amersham ECL Select (Cytiva) using the Fusion Solo system (Vilber Lourmat).

### TRF analysis by Southern blot

Isolated tissues were lysed in lysis buffer at 50 °C overnight (catalog no. K0512, Thermo Fisher Scientific) supplemented with 1 mg ml^−1^ proteinase K and RNase A (1:100 dilution, Sigma-Aldrich). Genomic DNA (gDNA) was extracted using equilibrated phenol-chloroform (Sigma-Aldrich) and chloroform-isoamyl alcohol extraction (Sigma-Aldrich). Equal amounts of gDNA were digested with the Rsal and HinFl enzymes (New England Biolabs) for 12 h at 37 °C. After digestion, samples were loaded on a 0.6% agarose gel, in 0.5% Tris/Borate/EDTA buffer and run on a CHEF-DRII pulse field electrophoresis apparatus (Bio-Rad Laboratories). The electrophoresis conditions were as follows: initial switch 1 s, final switch 6 s; voltage 4 V cm^−2^; at 4 °C for 20 h. Gels were then processed for Southern blotting using a 1.6-kb telomere probe, (TTAGGG)n, labeled with [α-32P]-dCTP.

### Telomerase activity assay

A real-time quantitative TRAP (Q-TRAP) assay was performed as described previously^67^. Protein extracts were obtained by adding 0.5% CHAPS to dissociate the tissue followed by 30 min of incubation on ice. Samples were centrifuged (16,000*g* for 20 min at 4 °C) and the supernatant was collected. Protein concentration was assessed with a Bradford assay, according to the manufacturer´s instructions. Then, 0.5 µg protein was added to the TRAP master mix (1× ABI SYBR Green, 10 mM EGTA, 100 ng ACX primer (5′-GCGCGGCTTACCCTTACCCTTACCCTAACC-3′), 100 ng TS (5′-AATCCGTCGAGCAGAGTT-3′), primer and RNase-free water up to 25 µl in a 96-well plate and incubated for 30 min at 28 °C in the dark. Real-time PCR was performed with a StepOnePlus Real-Time PCR System (Thermo Fisher Scientific): 95 °C for 10 min; 40 cycles at 95 °C for 15 s; and at 60 °C for 60 s. Each sample was performed in triplicate. As a negative control, samples were incubated with 1 mg RNase for 20 min at 37 °C. A standard curve for telomerase activity was obtained using 1:5 serial dilutions of HeLa extract. Data are presented as relative telomerase activity units, which was calculated according to the following formula: 10^((Ct sample]−Y_int_)/slope).

### Real-time qPCR and RNA-seq

Zebrafish were killed by lethal dose of 1 g l^−1^ of MS-222 and each tissue (gut, testes and kidney marrow) were dissected and immediately snap-frozen in liquid nitrogen. RNA extraction was performed by disrupting individual tissues with a pestle in TRIzol followed by chloroform extraction. The quality of RNA samples was assessed with a BioAnalyzer (Agilent Technologies). Retrotranscription into cDNA was performed using the QuantiTect Reverse Transcription Kit (QIAGEN).

qPCR was performed using the FastStart Universal SYBR Green Master mix (Roche) and a 7900HT Fast Real-Time PCR Detection System (Thermo Fisher Scientific). qPCR was carried out in triplicate for each cDNA sample. Relative mRNA expression was normalized against *rps11* mRNA expression using the 2^−ΔΔCT^ method as compared to the control condition. Primer sequences are listed in the Supplementary Information.

RNA-seq was performed by the Beijing Genomics Institute using, for each condition, biological triplicates, each consisting of a pool of two individual tissues. DNase-treated total RNA samples were enriched for mRNAs using oligo(dT) magnetic beads. In turn, mRNAs were fragmented into 200-bp fragments and the first strands of cDNAs were synthesized using random hexamers. To generate the library products, double-stranded cDNA from the second strand synthesis was purified using magnetic beads followed by A-tailing and RNA adapter ligation. The library was amplified with phi29 to make a DNA nanoball (DNB) that had more than 300 copies of each molecule. Paired-end, 150-bp reads were sequenced via combinatorial Probe-Anchor Synthesis on the DNBseq platform; 100 M clean reads per sample were generated. Raw data with adapter sequences or low-quality sequences were filtered using the SOAPnuke software developed by the Beijing Genomics Institute.

RNA-seq reads were analyzed via an internal pipeline for transcript quantification, normalization and comparison. Briefly, the human reference genome assembly vGRCh38 (retrieved from http://www.ensembl.org) and gencode annotation v.37 (retrieved from https://www.gencodegenes.org/) were processed with gffread v.0.12.2 to extract the human reference transcriptome. Based on this extracted reference transcriptome, Salmon v.1.4 was used to perform transcript quantification via quasi-mapping. RUVSeq v.1.20.0 was used for data transformation by rlog and data normalization by replicates. DESeq2 v.1.26.0 was used for differentially expressed gene (DEG) analysis. A false discovery rate (FDR) cutoff of 0.1 was explored for the DEG analysis (Supplementary Data).

The pathway enrichment analysis was performed using a GSEA approach, implemented using the Broad Institute’s GSEA software^68^^,^^69^. The enrichment was run using the hallmark geneset, retrieved from the Molecular Signature Database^68^^,^^70^, as well as the Fridman senescence UP geneset^71^ and Reactome’s SASP geneset (https://reactome.org/PathwayBrowser/#/R-HSA-2559582). Before enrichment, all geneset genes were mapped to zebrafish orthologs using the Ensembl’s BioMart database (Ensembl release 107, 2022). The GSEA parameters were set as follows: permutations = 1,000, permutation type = gene_set. Significant enrichment results were genesets with nominal *P* values and an FDR <0.05. Borderline significance (to indicate the directionality of a pathway) was set at nominal *P* values <0.05 and an FDR <0.25.

### Metagenomics

gDNA was extracted from the gut of sibling fish as described for the TRF analysis. The V3–V4 hypervariable regions of bacterial 16S rRNA genes were amplified by PCR with the Phusion High-Fidelity PCR mastermix (New England Biolabs) using the primer described previously^60^. PCR products were mixed at equal density ratios and purified with the Gel Extraction Kit (QIAGEN). Sequencing libraries were generated using the NEBNext Ultra DNA Library Prep Kit and sequenced on an Illumina NovaSeq 6000 paired-end platform to generate 250 bp paired-end raw reads. Sequence analysis was performed using the UPARSE software with all effective tags. Sequences with 97% or more similarity were assigned to the same operational taxonomic units (OTUs). Representative sequences for each OTU were screened for further annotation. For each representative sequence, the Mothur software was applied against the SILVA Small Subunit rRNA database for species annotation at each taxonomic rank (threshold: 0.8–1). QIIME and R were used to calculate α and β diversity metrics and generate plots. PCoA was performed to get principal coordinates and visualize complex, multidimensional data.

### Metabolomics

Each frozen gut sample was homogenized in 600 µl of methanol (HPLC grade, Merck Millipore) and incubated overnight at −20 °C. Tubes were vortexed and incubated overnight at −20 °C for protein precipitation. After centrifugation, the supernatants were removed, dried using a SpeedVac concentrator (Savant SVC100H, Thermo Fisher Scientific), resuspended in 80 µl of a 20:80 acetonitrile-H_2_O mixture (HPLC grade, Merck Millipore) and stored at −20 °C until use for the metabolomics analysis.

Chromatographic analysis was performed using a Dionex UltiMate 3000 HPLC system coupled to a chromatographic column (Phenomenex Synergi 4 µm Hydro-RP 80 Å 250 × 3.0 mm) set at 40 °C and a flow rate of 0.9 ml min^−1^. The gradients of mobile phases (mobile phase A: 0.1% formic acid in water and mobile phase B: 0.1% formic acid in acetonitrile) were performed for a total of 25 min. Mass spectrometry analysis was carried out on an Exactive Plus Benchtop Orbitrap mass spectrometer (Thermo Fisher Scientific). The heated electrospray ionization source (HESI II) was used in positive and negative ion modes. The instrument was operated in full-scan mode from 67 to 1,000 *m*/*z*. The post-treatment of data was performed using MZmine2 v.2.39 (http://mzmine.github.io/). Metabolites were identified using the Human Metabolome Database v.5.0 (http://www.hmdb.ca). We only used ions identified as (M + H)^+^ adducts in the positive mode and (M-H)^−^ adducts in the negative mode and ions found in all the samples after gap filling. For dataset denoising, only ions with average peak intensities greater than 10 × 10^5^ were considered.

### Statistics and reproducibility

Graphs and statistical analyses were performed in Prism 8 (GraphPad Software). For multiple comparisons, a one-way analysis of variance (ANOVA) with Tukey’s post hoc correction was used for normally distributed data and a Kruskal–Wallis test with a Dunn’s post hoc test was used for data that did not meet normality. A critical value for significance of *P* < 0.05 was used throughout the study. For the survival analysis, log-rank tests were performed with Prism8 to determine statistical differences of the survival curves.

Untargeted metabolomics analysis of gut samples was processed using statistical analysis (one-factor) modules proposed by MetaboAnalyst v.5.0 (https://www.metaboanalyst.ca). For each comparison, peak intensities were log-transformed. Clustering analysis was performed using PCA, PLS-DA and heatmap tools provided by MetaboAnalyst.

Statistical parameters and methods are reported in the figure legends. No statistical method was used to predetermine sample size. Sample sizes were chosen according to professional standards of the field for individual assays. Outlier identification was pre-established and performed using the Tukey’s method. Reported results were acquired using independent fish that were randomly collected for each group. (The number of fish used for each experiment is specified in each figure legend.) Except for the lifespan experiments, the investigators were not blinded to allocation during the experiments or data collection and outcome assessment or analysis.

### Ethics statement

The zebrafish work was conducted according to local and international institutional guidelines and was approved in France by the Animal Care Committee of the Institute for Research on Cancer and Aging, Nice, the regional (CIEPAL Côte d’Azur no. 697) and national (French Ministry of Research no. 27673-2020092817202619) authorities and in Portugal by the Ethics Committee of the Instituto Gulbenkian de Ciência and approved by the competent Portuguese authority (Direcção Geral de Alimentação e Veterinária; approval no. 0421/000/000/2015).

### Reporting summary

Further information on research design is available in the Nature Portfolio Reporting Summary linked to this article.

## Supplementary information

Supplementary Information Supplementary information list of primers used in RT–qPCR expression analysis.

Reporting Summary

Supplementary Data Supplementary Tables 1–6 Differentially expressed genes.

## Data Availability

Data generated or analyzed during this study are included in this published article, its Supplementary Information and Source Data files. The raw RNA-seq reads generated in this study have been deposited to the Sequence Read Archive database under accession no. PRJNA937311. Data used for the GSEA can be found online at https://github.com/maliabird17/El-Mai-GSEA-Analysis. All other data pertaining to this study are available from the corresponding author upon reasonable request.

## Source data

Source Data Fig. 1 Statistical source data.
Source Data Fig. 2 Statistical source data.
Source Data Fig. 3 Statistical source data.
Source Data Fig. 4 Statistical source data.
Source Data Fig. 5 Statistical source data.
Source Data Fig. 6 Statistical source data.
Source Data Fig. 7 Statistical source data.
Source Data Fig. 8 Statistical source data.
Source Data Extended Data Fig. 1 Statistical source data.
Source Data Extended Data Fig. 2 Statistical source data.
Source Data Extended Data Fig. 3 Statistical source data.
Source Data Extended Data Fig. 5 Statistical source data.
Source Data Extended Data Fig. 6 Statistical source data.
Source Data Extended Data Fig. 7 Statistical source data.
Source data Fig. 1c and Extended Data Fig. 1j,o Unprocessed Southern blots.
Source data Figs. 1e, 5b and 6b Unprocessed western blots.
