# Supplementary material for: Gut-specific telomerase expression counteracts systemic aging in telomerase-deficient zebrafish
Source: Nat Aging. 2023 May 4;3(5):567–84. doi: 10.1038/s43587-023-00401-5 (PMC10191862; doi:10.1038/s43587-023-00401-5)
Supplement: Supplementary file 1 — Supplementary information list of primers used in RT–qPCR expression analysis. [file 43587_2023_401_MOESM1_ESM.pdf]

nature aging

Article

https://doi.org/10.1038/s43587-023-00401-5

Gut-specific telomerase expression counteracts systemic aging in telomerase-deficient zebrafish

In the format provided by the authors and unedited

Supplementary Table – List of primers used in RT-qPCR expression analysis.

Gene name	Primer sequences
cdkn2a/b (p15/16)	forward - 5' GAGGATGAACTGACCACAGCA 3'<br>reverse - 5' CAAGAGCCAAAGGTGCGTTAC 3'
cdkn1a (p21)	forward - 5' CAGCGGGTTTACAGTTTCAGC 3'<br>reverse - 5' TGAACGTAGGATCCGCTTGT 3'
tnfa	forward - 5' AGGCAATTTCACTTCCAAGGC 3'<br>reverse - 5' GGTCCTGGTCATCTCTCCAGT 3'
tert	forward - 5' CGGTATGACGGCCTATCACT 3'<br>reverse - 5' TAAACGGCCTCCACAGAGTT 3'
tert transgene	forward - 5' GCATGTTAGAAGACTTCCTCTGC 3'<br>reverse - 5' TTCCTCTCCCAGAATCCCCC 3'
rps11	forward - 5' ACAGAAATGCCCCTTCACTG 3'<br>reverse - 5' GCCTCTTCTCAAAACGGTTG 3'
il-6	forward - 5' TCAACTTCTCCAGCGTGATG 3'<br>reverse - 5' TCTTTCCCTCTTTTCCTCCTG 3'
cyr 61	forward - 5' CCGTGTCCACATGTACATGGG 3'<br>reverse - 5' GGTGCATGAAAGAAGCTCGTC 3'
ctgf	forward - 5' ACTCCCCTCGTCAAAACACC 3'<br>reverse - 5' GGGACCGTATGTCTCCTCCT 3'
claudin-2	forward - 5' GCAACACCTCACTGCTGAAC 3'<br>reverse - 5' TTGCCCAGTAGGGGAGAAGA 3'
mmp2	forward - 5' AGCCTTAATGGTGATGGTCACAGCT 3'<br>reverse - 5' GTCCTTTTTGACCTCGCCGACTTTG 3'
mmp15a	forward - 5' GGGTCATGCTCTGGGGTTGG 3'<br>reverse - 5' AGTGGTGACAGTCTCTGGAGATCCA 3'
tgfb1b	forward - 5' ACCCAGTTCAGCACACCATAG 3'<br>reverse - 5' TCGAAACTCGGCCTGGTAGA 3'
tgfb5	forward - 5' TACGCCAAAGAGGTGCAGAG 3'<br>reverse - 5' AGTACGGACTCTGAGGACCC 3'
