# Supplementary material for: Gut-specific telomerase expression counteracts systemic aging in telomerase-deficient zebrafish
Source: Nat Aging. 2023 May 4;3(5):567–84. doi: 10.1038/s43587-023-00401-5 (PMC10191862; doi:10.1038/s43587-023-00401-5)
Supplement: Supplementary file 2 — Reporting Summary [file 43587_2023_401_MOESM2_ESM.pdf]

nature portfolio

Corresponding author(s): Miguel Godinho Ferreira

Last updated by author(s): Feb 20, 2023

Reporting Summary

Nature Portfolio wishes to improve the reproducibility of the work that we publish. This form provides structure for consistency and transparency in reporting. For further information on Nature Portfolio policies, see our Editorial Policies and the Editorial Policy Checklist.

Statistics

For all statistical analyses, confirm that the following items are present in the figure legend, table legend, main text, or Methods section.

n/a	Confirmed	
☐	☒	The exact sample size (*n*) for each experimental group/condition, given as a discrete number and unit of measurement
☐	☒	A statement on whether measurements were taken from distinct samples or whether the same sample was measured repeatedly
☐	☒	The statistical test(s) used AND whether they are one- or two-sided<br>*Only common tests should be described solely by name; describe more complex techniques in the Methods section.*
☐	☒	A description of all covariates tested
☐	☒	A description of any assumptions or corrections, such as tests of normality and adjustment for multiple comparisons
☐	☒	A full description of the statistical parameters including central tendency (e.g. means) or other basic estimates (e.g. regression coefficient) AND variation (e.g. standard deviation) or associated estimates of uncertainty (e.g. confidence intervals)
☐	☒	For null hypothesis testing, the test statistic (e.g. *F*, *t*, *r*) with confidence intervals, effect sizes, degrees of freedom and *P* value noted<br>*Give P values as exact values whenever suitable.*
☒	☐	For Bayesian analysis, information on the choice of priors and Markov chain Monte Carlo settings
☒	☐	For hierarchical and complex designs, identification of the appropriate level for tests and full reporting of outcomes
☒	☐	Estimates of effect sizes (e.g. Cohen's *d*, Pearson's *r*), indicating how they were calculated

Our web collection on statistics for biologists contains articles on many of the points above.

Software and code

Policy information about availability of computer code

Data collection	Applied Biosystems 7900HT Fast Real-Time PCR System Software SDS (v2.4) for RT-qPCR; Deltavision Softworx Software (v7.0.0) for immunofluorescence image acquisition; Leica Application Suite LAS software (v3.8) for histological image acquisition; Typhoon FLA9000 software (v1.2) for southern blot scans; Excalibur (v4.2, Thermo Fisher Scientific) was used for metabolomic acquisition; FusionCapt Advance Solo 4 v16.09b was used for western blots, StepOOne Software v2.3.
Data analysis	ImageJ (v2.0.0) for image analysis; Graphpad Prism (v8.0.2) and Microsoft Excel (v2108) for data and statistical analysis. For metabolomics, post-treatment of data was performed using the MZmine2 version 2.39 (http://mzmine.github.io/). Metabolites were identified using the Human Metabolome Database version 5.0 (http://www.hmdb.ca). Untargeted metabolomic analysis were processed using statistical analysis [one factor] modules proposed by MetaboAnalyst 5.0 (https://www.metaboanalyst.ca). For transcriptomics analysis, reference genome assembly and gencode annotation were processed by gffread v0.12.2 to extract reference transcriptome. Based on this extracted reference transcriptome, Salmon v1.4 was used to perform transcript quantification via quasi-mapping. RUVseq v1.20.0 was used for data transformation by "rlog" and data normalization by replicates. DESeq2 v1.26.0 was used for differentially expressed gene (DEG) analysis. The false discovery rate (FDR) cutoffs of both 0.1 and 0.05 were explored for the DEG analysis. Based on the resulting DEG candidate gene lists, clusterProfiler v4.0 was employed for Gene Ontology (GO) analysis and Gene Set Enrichment Analysis (GSEA), based on which KEGG pathway enrichment analysis were further performed.

For manuscripts utilizing custom algorithms or software that are central to the research but not yet described in published literature, software must be made available to editors and reviewers. We strongly encourage code deposition in a community repository (e.g. GitHub). See the Nature Portfolio guidelines for submitting code & software for further information.

Data

Policy information about availability of data

All manuscripts must include a data availability statement. This statement should provide the following information, where applicable:

- Accession codes, unique identifiers, or web links for publicly available datasets
- A description of any restrictions on data availability
- For clinical datasets or third party data, please ensure that the statement adheres to our policy

All data generated or analysed during this study are included in this published article and its supplementary information files.

Field-specific reporting

Please select the one below that is the best fit for your research. If you are not sure, read the appropriate sections before making your selection.

☒ Life sciences ☐ Behavioural & social sciences ☐ Ecological, evolutionary & environmental sciences

For a reference copy of the document with all sections, see nature.com/documents/nr-reporting-summary-flat.pdf

Life sciences study design

All studies must disclose on these points even when the disclosure is negative.

Sample size	All sample sizes were based on standard protocols and previous publications from our lab (for example, see PMID: 32427102, PMID: 23349637, PMID: 26789415) . No statistical test was performed to predetermine sample size.
Data exclusions	Outlier identification was pre-established and performed using Tukey's method.
Replication	All experiments were performed using independent biological replicates. Number of replicates (N) used in each experiment are described in figure legends.<br>RNA sequencing experiment include biological triplicates consisting for each of a pool of two individual tissues.
Randomization	Reported results were acquired using independent fish that were randomly collected for each group (number of fish used for each experiment is specified in each figure legend).
Blinding	Except for lifespan experiments, the investigators were not blinded to allocation during the experiments and/or data collection and outcome assessment or analysis.

Reporting for specific materials, systems and methods

We require information from authors about some types of materials, experimental systems and methods used in many studies. Here, indicate whether each material, system or method listed is relevant to your study. If you are not sure if a list item applies to your research, read the appropriate section before selecting a response.

Materials & experimental systems

n/a	Involved in the study	
☐	☒	Antibodies
☒	☐	Eukaryotic cell lines
☒	☐	Palaeontology and archaeology
☐	☒	Animals and other organisms
☒	☐	Human research participants
☒	☐	Clinical data
☒	☐	Dual use research of concern

Methods

n/a	Involved in the study	
☒	☐	ChIP-seq
☒	☐	Flow cytometry
☒	☐	MRI-based neuroimaging

Antibodies

Antibodies used	For immunofluorescence staining :<br>PCNA (Santa Cruz, #sc56, 1:50)<br>MPX (GeneTex, GTX128379, 1:50)<br>gH2AX (GeneTex, GTX127342, 1:50)<br>L-plastin (GeneTex, GTX124420, 1:100)<br>Alexa Fluor 488 goat anti-rabbit IgG (H+L) (#A11008, Invitrogen, 1:500)<br>Alexa Fluor 488 goat anti-mouse IgG (H+L) (#A11001, Invitrogen, 1:500)

For Western Blotting:
anti-p53 (ANASPEC-55342, 1:1000)
anti-Actin (Sigma A2066, 1:1000)

Validation

PCNA (Santa Cruz, #sc56) was validated by the manufacturer for immunofluorescence and used on zebrafish sections in PMID: 31450674 (IHC) and PMID: 29695612 (IF)
MPX (GeneTex, GTX128379) was validated by the manufacturer for immunofluorescence on zebrafish and used on zebrafish in PMID: 29666124 (IF) and PMID: 32678226 (IF)
gH2AX (GeneTex, GTX127342) was validated by the manufacturer for immunofluorescence on zebrafish and used on zebrafish in PMID: 35635436 (IF) and PMID: 35436990
L-plastin (GeneTex, GTX124420) was validated by the manufacturer for immunofluorescence on zebrafish and used on zebrafish in PMID: 30805338 and PMID: 35820929

Animals and other organisms

Policy information about studies involving animals; ARRIVE guidelines recommended for reporting animal research

Laboratory animals

The zebrafish telomerase mutant line tert AB/hu3430 (ZDB-FISH-150901-19562) was previously generated by N-Ethyl-N-nitrosourea (ENU) mutagenesis (PMID: 15602906).
The zebrafish Tg(Fabp2: LoxP-dsRed-loxP-tert-T2A-CFP) line was generated using Tol2-mediated transgenesis in AB strain (see methods).
Both lines were combined to produce experimental fish. Overall characterization of the different experimental genotypes was performed in F1 sibling animals at 9 months of age. Due to a male sex bias in our crosses, that affected mostly tert-/- progeny, we were unable to obtain significant numbers of females for analysis and so all of our data except survival analysis are restricted to males.

Wild animals

no wild animals were used in this study

Field-collected samples

No field-collection of samples was performed in this study

Ethics oversight

Zebrafish work was conducted according to local and international institutional guidelines and were approved in France by the Animal Care Committee of the IRCAN, the regional (CIEPAL Cote d'Azur #697) and national (French Ministry of Research #27673-2020092817202619) authorities and in Portugal by the Ethical Committee of the Instituto Gulbenkian de Ciência and approved by the competent Portuguese authority (Direcção Geral de Alimentação e Veterinária; approval number: 0421/000/000/2015).

Note that full information on the approval of the study protocol must also be provided in the manuscript.
