# Supplementary figures and images for: Gut-specific telomerase expression counteracts systemic aging in telomerase-deficient zebrafish
Source: Nat Aging. 2023 May 4;3(5):567–84. doi: 10.1038/s43587-023-00401-5 (PMC10191862; doi:10.1038/s43587-023-00401-5)

Gut:

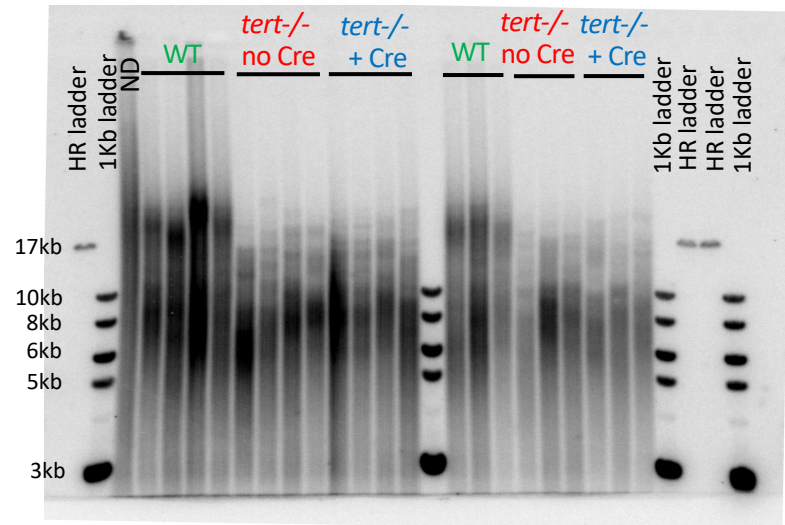

Testes:

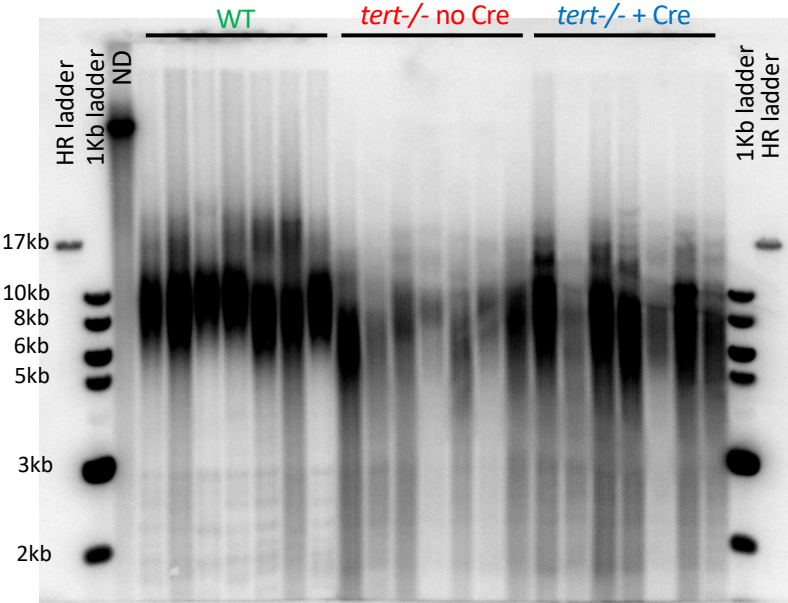

Kidney marrow :

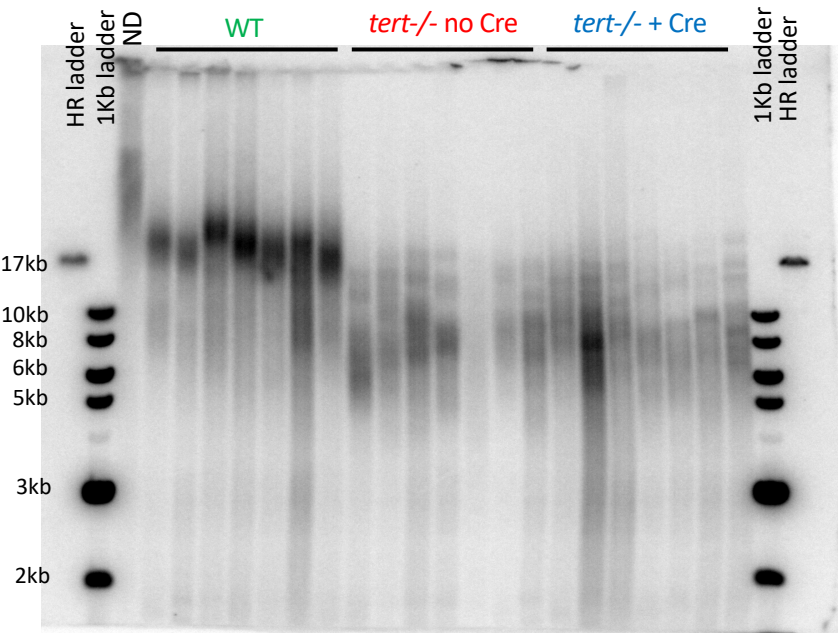

Supplement: Source data Fig. 1c and Extended Data Fig. 1j,o — Unprocessed Southern blots. [file 43587_2023_401_MOESM18_ESM.pdf]
