# Supplementary material for: Gut-specific telomerase expression counteracts systemic aging in telomerase-deficient zebrafish
Source: Nat Aging. 2023 May 4;3(5):567–84. doi: 10.1038/s43587-023-00401-5 (PMC10191862; doi:10.1038/s43587-023-00401-5)
Supplement: Source data Figs. 1e, 5b and 6b — Unprocessed western blots. [file 43587_2023_401_MOESM19_ESM.pdf]

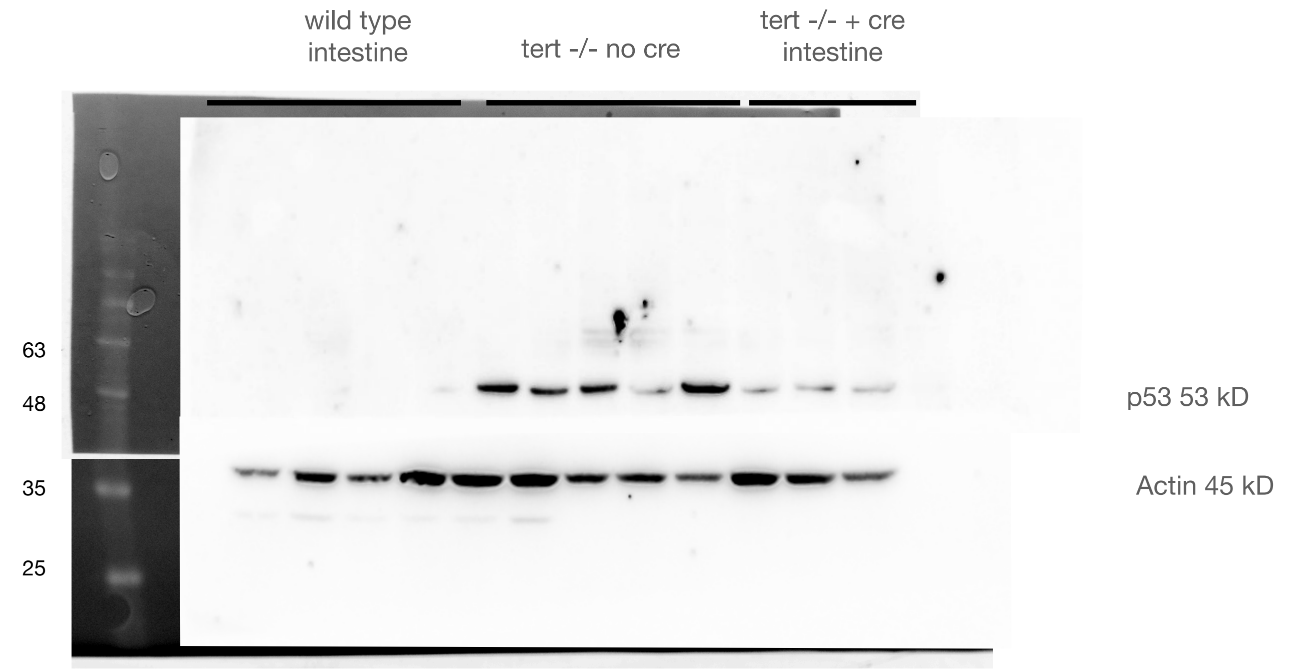
wild type intestine
tert -/- no cre
tert -/- + cre intestine
63
48
35
25
p53 53 kD
Actin 45 kD

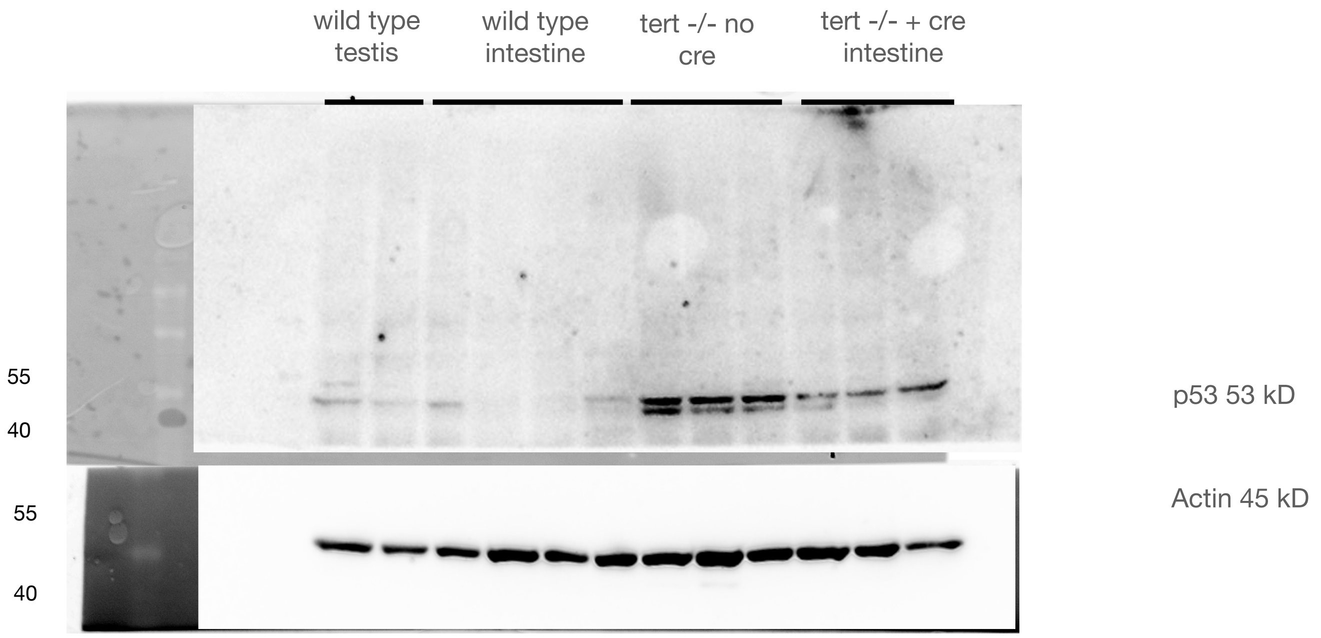
wild type testis
wild type intestine
tert -/- no cre
tert -/- + cre intestine
55
40
p53 53 kD
55
40
Actin 45 kD

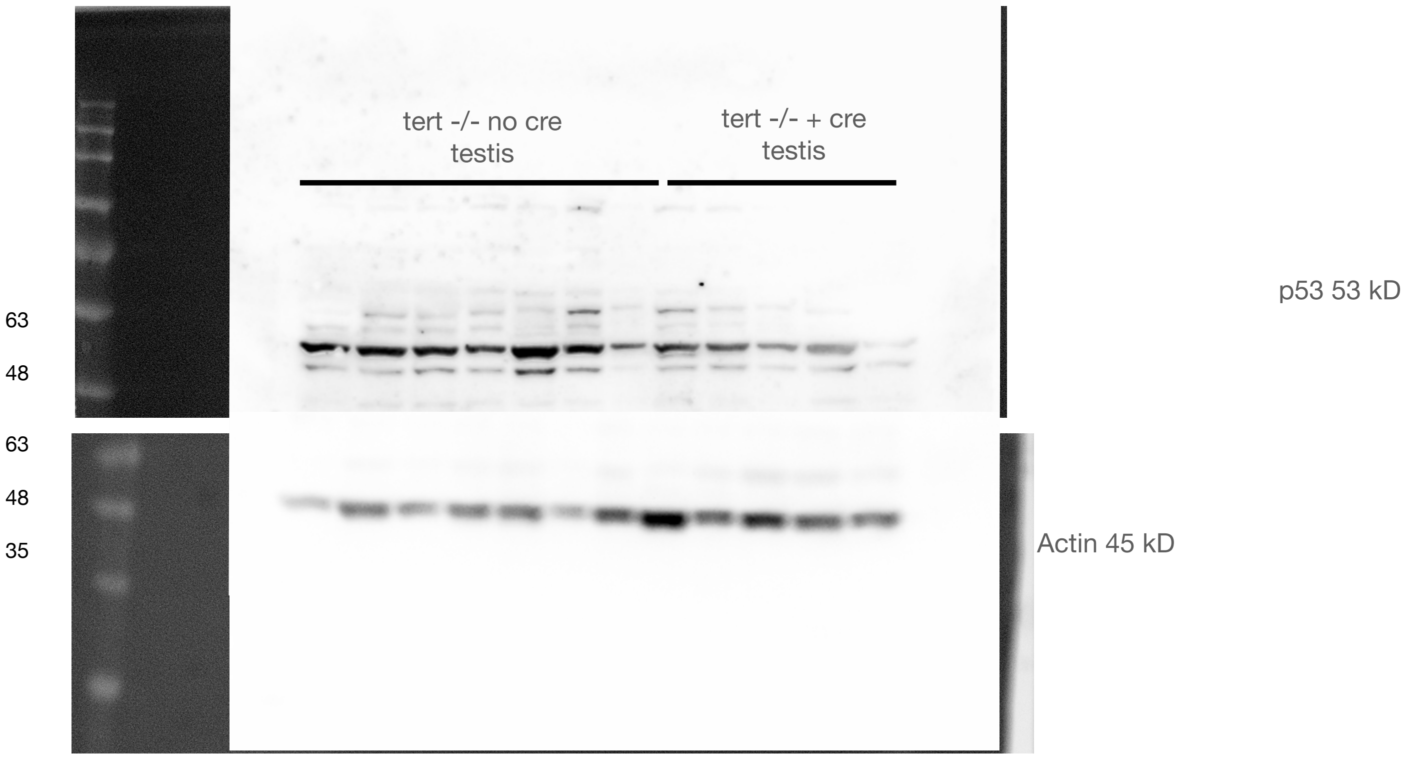
tert -/- no cre testis
tert -/- + cre testis
63
48
p53 53 kD
63
48
35
Actin 45 kD

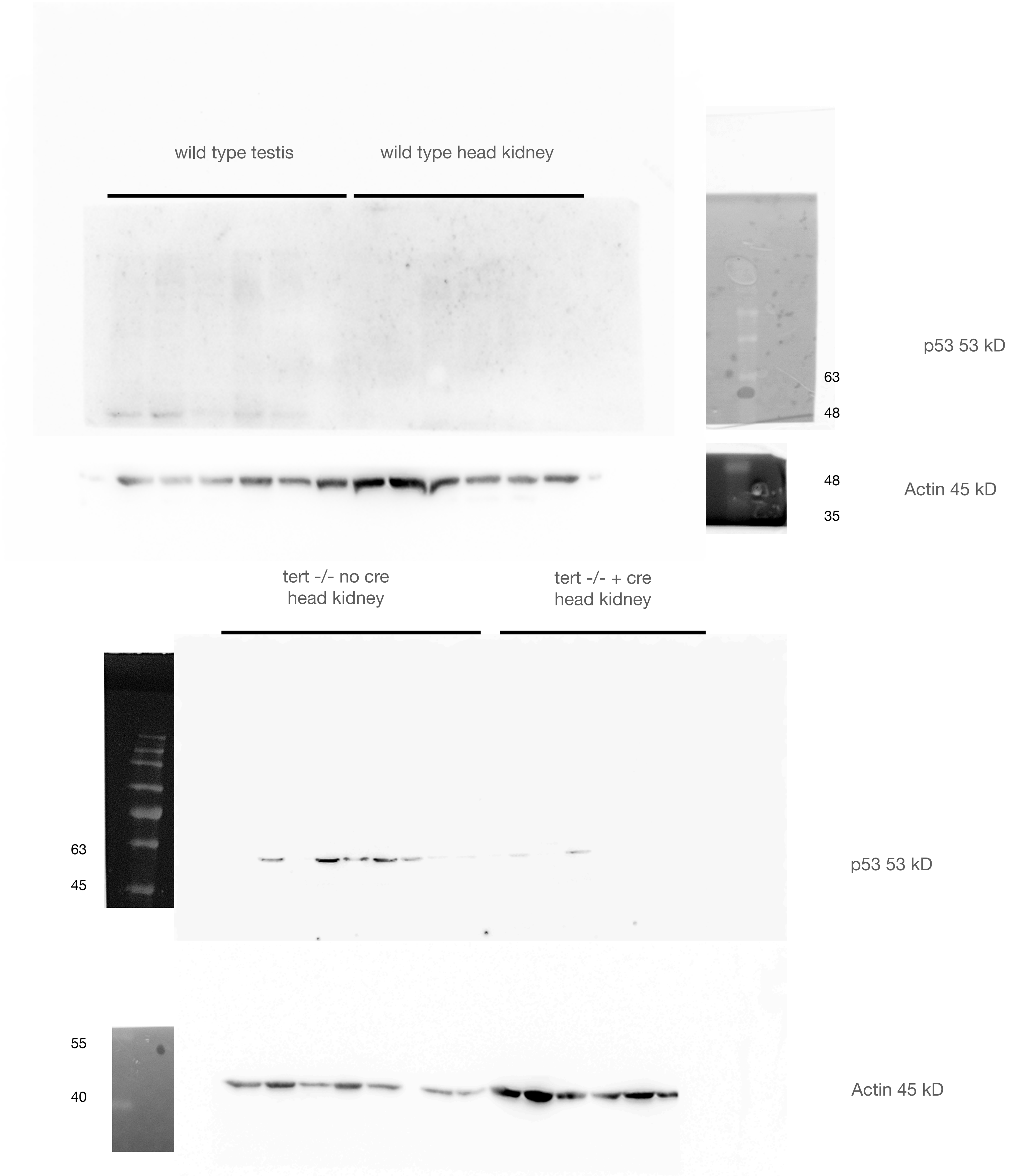

wild type testis
wild type head kidney
p53 53 kD
63
48
48
35
Actin 45 kD
tert -/- no cre
head kidney
tert -/- + cre
head kidney
63
45
p53 53 kD
55
40
Actin 45 kD
